# Binary Phase Diagrams of Coordination Polymers with Eutectic Behaviors

**DOI:** 10.1021/jacs.4c15317

**Published:** 2025-02-03

**Authors:** Karnjana Atthawilai, Hiroyasu Tabe, Kotaro Ohara, Kanokwan Kongpatpanich, Satoshi Horike

**Affiliations:** † Department of Materials Science and Engineering, School of Molecular Science and Engineering, Vidyasirimedhi Institute of Science and Technology, Rayong 21210, Thailand; ‡ Institute for Integrated Cell-Material Sciences, Institute for Advanced Study, Kyoto University, Yoshida-Hommachi, Sakyo-ku, Kyoto 606-8501, Japan; § Department of Synthetic Chemistry and Biological Chemistry, Graduate School of Engineering, Kyoto University, Katsura, Nishikyo-ku, Kyoto 615-8510, Japan; ∥ Department of Chemistry, Graduate School of Science, Kyoto University, Kitashirakawa-Oiwakecho, Sakyo-ku, Kyoto 606-8502, Japan

## Abstract

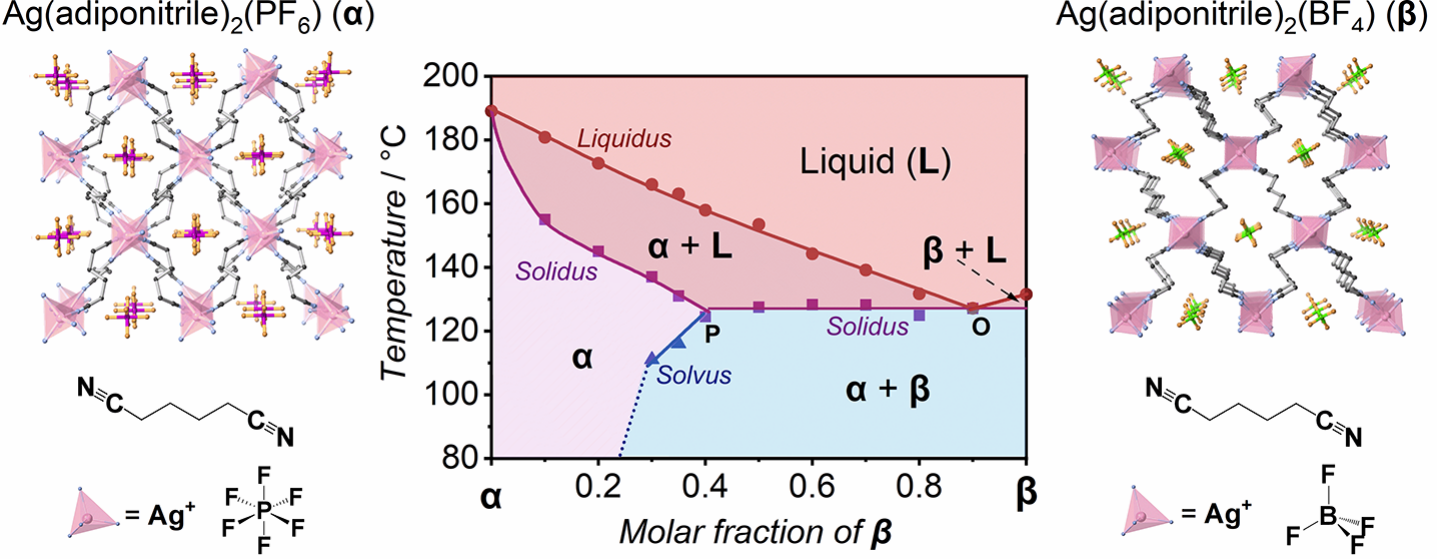

We demonstrated the construction of binary phase diagrams of coordination polymer crystals by using their reversible solid–liquid transition (melting and crystallization) behaviors. Three types of binary phase diagrams of Ag^+^-based coordination polymers were made and the origins of each diagram formation were investigated. All diagrams showed eutectic phenomena induced by ligand exchange reactions at the interface of the two constituents. Solid solution formation was observed when we used two crystals with similar structures and coordination geometries, and the reaction was driven by both the anion and ligand exchange processes. The optimal binary compounds found in the phase diagrams were applicable as a latent heat storage material working at 100 °C with high energy recovery efficiency, stability in phase-transition cycles, and large hysteresis of melting/crystallization.

## Introduction

Creating binary systems of metals or ceramics is a fundamental strategy for controlling the properties and functionalities of materials. Steel (Fe–C) and brass (Cu–Zn) are classical metallic alloys with formability and corrosion resistance.^1−3^ Al_2_O_3_–La_2_O_3_ and CeO_2_–ZrO_2_ are ceramic catalysts with high thermal stability.^4^ The properties of binary systems are dominated by both compositions and microstructures such as solid solutions, intermetallic compounds, and alloys. The formation of the binary systems largely influences thermal properties (e.g., melting and crystallization temperatures, thermal expansion) and mechanical properties (e.g., modulus, toughness).^5−8^

Coordination polymers (CPs) and metal–organic frameworks (MOFs) comprise metal ions and bridging ligands to form extended structures, usually in highly crystalline states. It has been long recognized that CP/MOFs do not exhibit a stable liquid state at elevated temperatures. On the other hand, recent reports suggest that several classes of CP/MOFs exhibit reversible crystal-to-liquid transformation by heating, and the behavior opens a new direction for materials engineering.^9−14^ There is a report on the single phase diagram of Zn(Im)_1.75_(bIm)_0.25_ (ZIF-62, Im: imidazolate, bIm: benzimidazolate) whose melting temperature (*T*_m_) at 1 bar is 430 °C.^15^ The diagram describes phases found at a given pressure and temperature; two amorphous phases exist, where the liquid is at 2 GPa and 350 °C. This report is only an example of an approaching phase diagram of CP/MOFs, and no attempt has been made to produce a binary phase diagram.

The number of reports of organic–inorganic hybrid materials such as CP/MOFs and hybrid perovskites showing reversible melting and recrystallization behavior continues to increase.^16−22^ It is important to use these compounds as a platform to realize the construction of binary phase diagrams and discover previously unknown phases and physical properties. To date, binary phase diagrams of hybrid perovskites are reported.^23−28^ In this work, we attempted for the first time to construct binary phase diagrams of CP/MOFs and investigate the origins of the diagram formations. Reversible melting and crystallization without forming metastable states, such as glass, are essential to the making of phase diagrams. The labile character of the nitrile group plays an important role in melting and crystallization due to low viscosity and melting temperature (*T*_m_) compared with other CPs showing crystal melting, such as ZIFs.^19,29,30^ The dinitrile CPs showing crystal melting are reported using *d*^10^ Ag^+^ ions. In this context, CPs consisting of alkyldinitrile and Ag^+^ ions are candidates as constituents of binary systems because the crystal similarity of Ag^+^-alkyldinitrile CPs is well discussed in the literature.^31^ We employed five compounds, all composed of Ag^+^ ions and dinitrile ligands: Ag(GN)_2_(BF_4_) (**1**, GN = glutaronitrile), Ag(PN)_2_(BF_4_) (**2**, PN = pimelonitrile), Ag(AN)_2_(BF_4_) (**3**, AN = adiponitrile), Ag(AN)(OTf) (**4**, OTf = trifluoromethanesulfonate ion), and Ag(AN)_2_(PF_6_) (**5**). We constructed three types of binary phase diagrams and found the behaviors of eutectic melting and solid solution formation depending on the combinations of crystal structures and compositions. It was found that the dynamic exchange of ligands and anions was also the key to show eutectic and solid solution formation. One of the optimal binary compounds showed promising latent heat storage properties attributed to the large hysteresis by supercooling, the working temperature, and high energy recovery efficiency.

## Experimental Section

### Materials

All chemicals were used as delivered. Silver tetrafluoroborate (AgBF_4_), silver trifluoromethanesulfonate (AgOTf), silver hexafluorophosphate (AgPF_6_), and benzene were purchased from FUJIFILM-Wako Pure Chemical Industries Corporation (Japan). Glutaronitrile (GN), pimelonitrile (PN), and adiponitrile (AN) were purchased from Tokyo Chemical Industry Co., Ltd. Indium powder (99.99%) was purchased from Sigma-Aldrich Co., LLC (USA). Purified water was supplied by Milli-Q (Merck-Millipore System Corporation, USA), where the electronic conductance was 18.2 MΩ cm.

### Synthesis of CPs

Ag(GN)_2_(BF_4_) (**1**) was synthesized as follows. Benzene (20 mL) containing AgBF_4_ (97 mg, 0.5 mmol) and GN (94 mg, 1 mmol) was magnetically mixed for 5 min and transferred to a Teflon tube followed by heating at 120 °C under solvothermal conditions. After cooling to room temperature for 2 days, colorless crystals were obtained. The crystals were washed with benzene three times and dried *in vacuo* to obtain rodlike crystals. The obtained crystals have powder X-ray diffraction (PXRD) patterns similar to those simulated from the crystal structure (Figure S1). The CN stretching band (ν_CN_) found in Fourier transform infrared (FT-IR) spectroscopy: 2273 cm^–1^ (Figure S2); decomposition temperature (*T*_d_) determined by thermogravimetric analysis (TGA, Figure S3): 161 °C; melting temperature (*T*_m_) determined by differential scanning calorimetry (DSC, Figure S4): 147 °C; crystallization temperature determined by DSC (*T*_c_): 143 °C (Table S1).

Ag(PN)_2_(BF_4_) (**2**) was synthesized from AgBF_4_ (97 mg, 0.5 mmol) and PN (122 mg, 1 mmol). ν_CN_: 2277 cm^–1^, *T*_m_: 120 °C, *T*_c_: 115 °C, *T*_d_: 156 °C. Ag(AN)_2_(BF_4_) (**3**) was synthesized from AgBF_4_ (97 mg, 0.5 mmol) and AN (108 mg, 1 mmol). ν_CN_: 2277 cm^–1^, *T*_m_: 132 °C, *T*_c_: 128 °C, *T*_d_: 150 °C. Endothermic and exothermic peaks found at 40 and 37 °C result from solid–solid phase transition as suggested by PXRD (Figure S5). Ag(AN)(OTf) (**4**) was synthesized from AgOTf (128 mg, 0.5 mmol) and AN (108 mg, 1 mmol). ν_CN_: 2277 cm^–1^, *T*_m_: 148 °C, *T*_c_: 134 °C, *T*_d_: 173 °C. Ag(AN)_2_(PF_6_) (**5**) was synthesized from AgPF_6_ (127 mg, 0.5 mmol) and AN (108 mg, 1 mmol). ν_CN_: 2277 cm^–1^, *T*_m_: 189 °C, *T*_c_: 180 °C, *T*_d_: 213 °C. All compounds have powder X-ray diffraction (PXRD) patterns similar to those simulated from the crystal structures (Figure S1).

### Preparation of Binary Compounds by Mechanical Mixing

**1** (176.1 mg, 0.46 mmol) and **2** (237.1 mg, 0.54 mmol) were weighed by an electric balance AUY220 (Shimadzu, Japan), and 10 zirconium oxide (ZrO_2_) balls (φ = 10 mm) were transferred to a ZrO_2_ jar under a N_2_ atmosphere. The sample was ball-milled at 400 rpm, which involves 5 min run and 5 min pause for six cycles. **1**_*x*_**2**_1–*x*_ with other ratios (*x* = 0.02, 0.05, 0.1, 0.2, 0.3, 0.33, 0.5, 0.67, 0.75, 0.9, 0.95), binary compounds consisting of **3** and **4** (**3**_*x*_**4**_1–*x*_, *x* = 0.1, 0.2, 0.3, 0.4, 0.5, 0.6, 0.7, 0.8, 0.9), and **3** and **5** (**3**_*x*_**5**_1–*x*_, *x* = 0.1, 0.2, 0.3, 0.4, 0.5, 0.6, 0.7, 0.8, 0.9) were obtained using the same procedure.

### Single-Crystal X-ray Structure Analysis of **5**

Single-crystal X-ray crystallographic data of **5** were collected at 293 K on a Rigaku XtaLAB AFC10 diffractometer (Rigaku, Japan, Mo-*K*α, λ = 0.71073 Å). CrysAlis^Pro^ software (Rigaku, Japan) was used to determine the unit cell parameters, data reduction, scaling, and absorption corrections. The structure was solved in the orthorhombic *Pnna* space group by intrinsic phasing with SHELXT and refined by the full-matrix least-squares method on *F*^2^ using SHELXL as implemented in OLEX.^32−34^ The obtained crystal structure of **5** was registered in the Cambridge Structural Database (CSD) No. 2286694 (Table S2).

### Powder X-ray diffraction (PXRD) Analysis

PXRD patterns were recorded on a MiniFlex 600 diffractometer (Rigaku, Japan). Incident X-ray radiation was produced by a Cu X-ray tube operating at 40 kV and 15 mA with Cu Kα radiation (λ = 1.54 Å). The scan rate was 10° min^–1^ within 2θ = 5–50°. Variable-temperature (VT) PXRD patterns were recorded on a MiniFlex 600 diffractometer equipped with a BTS500 (Anton Paar, Austria) heating stage under a N_2_ flow. The scan rate was 2° min^–1^ from 2θ = 5–50°. Peaks of 2θ = 38.5 and 46.3° derived from silver(0) were not observed in all of the measurements, while broad peaks of the sample tray were observed at 2θ = 7°. The simulated patterns were obtained using Mercury 4.0 and structure data of **1** (180608), **2** (180616), **3** (180611), and **4** (180615) registered in the CSD.^31,35^

### X-ray Total Scattering and Pair Distribution Function (PDF) Analyses

**3**, **5**, and **3**_0.2_**5**_0.8_ were sealed in borosilicate glass capillaries (φ = 1.0 mm). The scattering data were collected by four CdTe and two Ge detectors covering the *Q* range up to 25 Å at the BL04B2 beamline in SPring-8 (112.8 keV, λ = 0.110 Å). The scattering data were applied absorption, background, and Compton scattering corrections. The data were normalized to give the Faber*–*Ziman total structure factor (*S*(*Q*)) processed by absorption, background, and Compton scattering corrections.^36^ PDF was calculated by the Fourier transformation of *S*(*Q*) with a Lorch modification function by using Igor Pro.^37,38^

### Spectroscopic and Thermal Measurements

Fourier transform-infrared (FT-IR) spectroscopy was performed with a Nicolet Magna FT-IR spectrophotometer (Thermo Scientific, USA) equipped with a diamond ATR accessory and a heating unit. The resolution of the collected IR spectra was 2 cm^–1^. Scanning electron microscopy (SEM) images were obtained using a JSM-6500F (JEOL, Japan) scanning electron microscope operated at 15 kV. Thermogravimetry-differential thermal analyses (TG/DTA) were performed using a Rigaku TG8120 instrument (RIGAKU, Japan) under an Ar flow with the ramp rate of 10 °C min^–1^. Differential scanning calorimetry (DSC) experiments were carried out by a DSC 8500 Lab System instrument (PerkinElmer, USA) under an Ar flow using an aluminum sample folder and a lid with the scan rate of 10 °C min^–1^ unless other rates are noted. The uncertainties of the phase-transition temperature and Δ*H* are 0.03 and 0.07%, respectively, estimated from the cyclic DSC measurements of indium (99.99%). The scan range is below each *T*_d_ determined by TG-DTA, and we confirmed no weight loss during the DSC measurements. We used DSC data of the first heating–cooling cycle to determine *T*_m_ and *T*_c_ to avoid the effect of thermal history of samples. The first deviation from baselines represents an inset of an endothermic peak, and the intersection of baselines and final tangent lines represents an onset. Solidus and liquidus lines were depicted by plotting the inset temperatures, while solvus lines were depicted by plotting the onset temperatures.^39,40^

## Results and Discussion

### Binary Phase Diagrams of **1** and **2**

**Figure 1:**
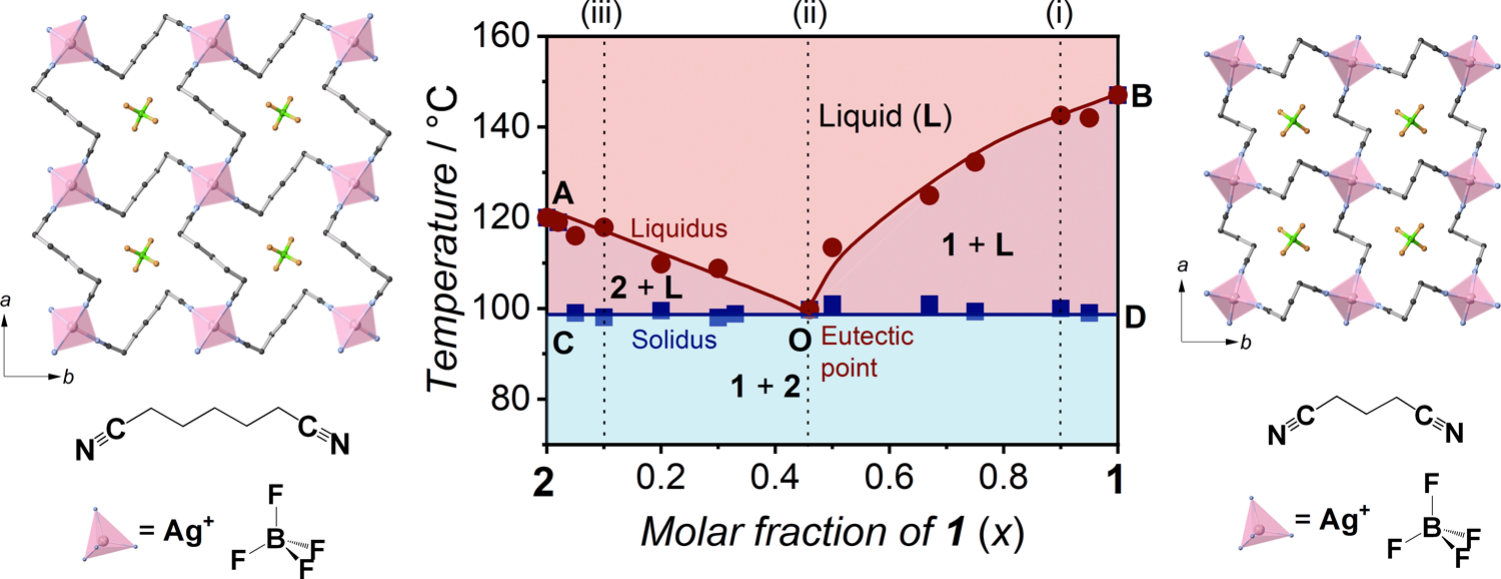
Binary phase diagram of Ag(GN)_2_(BF_4_) (**1**, right) and Ag(PN)_2_(BF_4_) (**2**, left) with crystal structures and components of **1** and **2**. C, N, Ag, B, and F atoms are black, blue, pink, green, and orange, respectively. Dashed lines marked as (i), (ii), and (iii) indicate the corresponding PXRD patterns shown in Figure S8.

Mechanical mixing is a general technique to obtain binary compounds in metallurgy.^1,2^ In this study, we define the mechanical mixing products of two crystals as binary compounds. Both **1** and **2** have four-coordinated Ag^+^ ions in tetrahedral geometries, two-dimensional (2D) structures of Ag^+^ ions and dicyanides, and BF_4_^–^ anions (Figure 1).^31^ The size difference of the 2D structures results from the length of the alkyl chains. Binary compounds prepared by mixing **1** and **2** are referred to as **1**_*x*_**2**_1–*x*_, where *x* (*x* = 0.02, 0.05, 0.1, 0.2, 0.3, 0.33, 0.46, 0.5, 0.67, 0.75, 0.9, and 0.95) represents the molar fraction of **1**. PXRD patterns of **1**_*x*_**2**_1–*x*_ involving peaks derived from **1** and **2** clarified that no chemical reaction and amorphization occurred in the mechanical mixing process (Figure S6A).

DSC curves of **1**_*x*_**2**_1–*x*_ have endothermic peaks in upscan due to their melting (Figure S7A). **1**_0.46_**2**_0.54_ has one endothermic peak at 100 °C. **1**_*x*_**2**_1–*x*_ in **1**-rich compositions (*x* > 0.46) have first endothermic peaks at 100 °C and second endothermic peaks (Table S3). For example, **1**_0.9_**2**_0.1_ has the second endothermic peak at 143 °C, which is lower than *T*_m_ of **1** (147 °C). We observed drops of the temperatures of second endothermic peaks of **1**_0.75_**2**_0.25_, **1**_0.67_**2**_0.33_, and **1**_0.5_**2**_0.5_ to 132, 125, and 113 °C, respectively, in **1**-rich compositions. The first endothermic peaks of **1**_*x*_**2**_1–*x*_ in **2**-rich compositions (*x* < 0.46) are 100 °C, and the temperatures of second endothermic peaks are lower than *T*_m_ of **2** (120 °C). For example, **1**_0.1_**2**_0.9_ has the first and second endothermic peaks at 100 and 118 °C, respectively.

**Figure 2:**
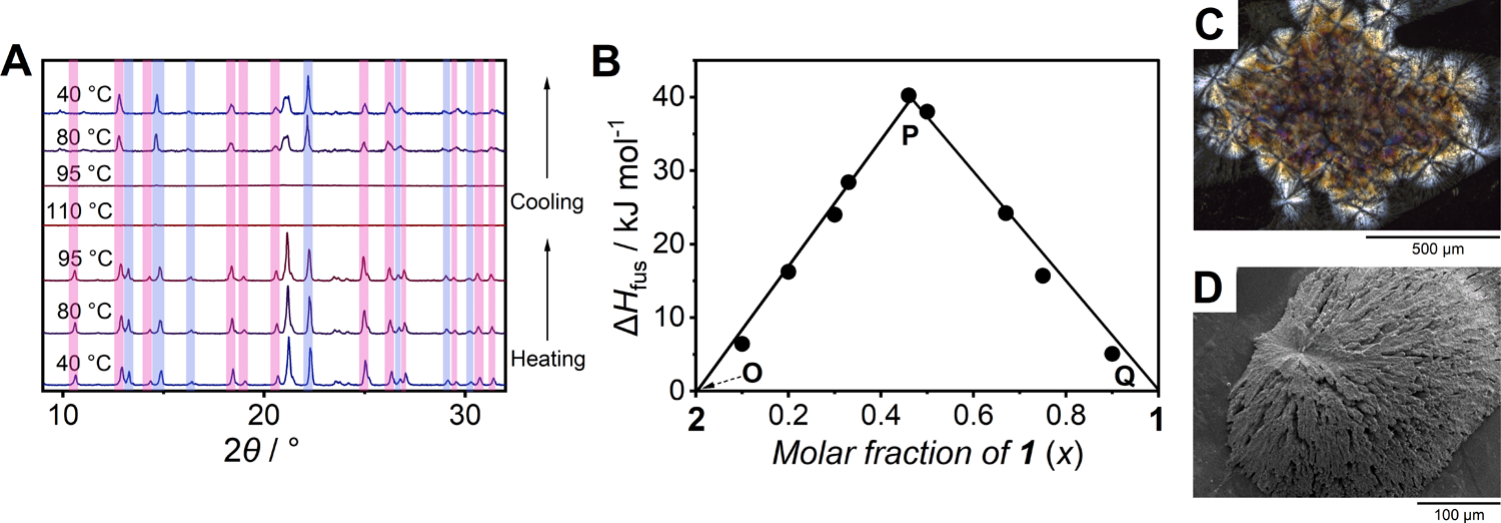
(A) PXRD patterns of **1**_0.46_**2**_0.54_ in the heating and cooling processes. Color masks represent diffraction peaks derived from **1** (blue) and **2** (pink), respectively. (B) Tamman’s diagram of **1**_*x*_**2**_1–*x*_. (C) Polarized optical microscopy and (D) SEM images of **1**_0.46_**2**_0.54_ after crystallization of the **1**_0.46_**2**_0.54_ liquid.

We measured variable-temperature (VT) PXRD for **1**_*x*_**2**_1–*x*_ (Figures 2A and S8). PXRD patterns of **1**_0.46_**2**_0.54_ at 40 and 80 °C consist of the patterns of **1** and **2** (composition shown as (ii) in Figures 1, 2A, and S8B). The intensity begins to decrease at 95 °C. The scattering pattern at 110 °C, which is above *T*_m_ (100 °C, by DSC), indicates that **1** and **2** simultaneously melt at 100 °C (Figure 2A). PXRD patterns of **1**_0.9_**2**_0.1_ at 40, 80, and 95 °C consist of peaks derived from **1** and **2** (composition shown as (i) in Figure 1, Figure S8A). **1**_0.9_**2**_0.1_ showed the pattern of **1** at 110 °C, which is above the temperature of first endothermic peak (100 °C by DSC), and a scattered pattern at 145 °C, which is above the temperature of second endothermic peak (143 °C by DSC, Figure S8A). The first and second endothermic peaks indicate the eutectic and equilibrium *T*_m_, respectively. Constituents in the eutectic ratio (**1**_0.46_**2**_0.54_) and excess **1** successively melt at eutectic and equilibrium *T*_m_, respectively, in **1**-rich compositions. VT-PXRD patterns of **1**_0.1_**2**_0.9_ indicate the disappearance of **1** and **2** peaks at 110 and 130 °C, respectively (Figure S8C). The temperatures of first (100 °C) and second (117 °C) endothermic peaks by DSC are the eutectic and equilibrium *T*_m_, respectively (composition shown as (iii) in Figure 1, Figure S8C).

We depicted a composition–temperature binary phase diagram of the **1**–**2** system (Figure 1). The phase diagram represents that the **1**–**2** system is a binary eutectic system showing no miscibility in the solid state but complete miscibility in the liquid state, like the Bi–Cd system.^41^ A horizontal line (CD) is a solidus line by plotting the eutectic *T*_m_ at 100 °C, also recognized as the eutectic temperature (*T*_e_). Two liquidus lines (AO and BO) are depicted by plotting the equilibrium *T*_m_ versus *x*, and they represent the depression of *T*_m_ of **2** (120 °C) and **1** (147 °C). AO and BO reach the intersection at O. O is the eutectic point corresponding to the eutectic composition (**1**_0.46_**2**_0.54_) and *T*_e_ (100 °C). Three phases, i.e., **1** solid, **2** solid, and the mixed liquid of **1** and **2** exist at O. All phases are thermodynamically stable, and no metastable phases, such as glass, appear in the diagram. We made a theoretical binary phase diagram using the Schroeder–van Laar equation (Figure S9).^42,43^ The estimated eutectic ratio and *T*_e_ are **1**_0.38_**2**_0.62_ and 102 °C, respectively. The equation links the interaction parameter (χ), *T*_m_, and fusion enthalpy (Δ*H*_fus_) of two constituents. The difference between the experimental and estimated eutectic ratio and *T*_e_ results from specific interactions observed in **1**_*x*_**2**_1–*x*_, which are not involved in the equation, such as the Ag–N coordination in the liquid state.^44^

Tamman’s diagram is useful for discussing the eutectic point and solid solution formation of binary compounds based on thermodynamic parameters.^45,46^ The diagram represents the fusion enthalpy (Δ*H*_fus_) at *T*_e_ as a function of the molar fraction of constituents. Δ*H*_fus_ increases as the molar fraction comes close to the eutectic composition. Tamman’s diagram of **1** and **2** (Figure 2B) consists of PO and PQ lines, which are linear regressions of Δ*H*_fus_–*x* plots. The Δ*H*_fus_ maximum at *x* = 0.46 indicates that **1**_0.46_**2**_0.54_ is in the eutectic composition (Figure 2B and Table S4). PO and PQ lines crossing the composition axis at *x* = 0 and 1 mean no miscibility in the solid state in the **1**–**2** system.

The DSC measurements of **1**_0.46_**2**_0.54_ in seven heating–cooling cycles indicate the reversible melt and crystallization in the heating and cooling processes (Figure S10). The crystallization temperature (*T*_c_) is 80 °C due to supercooling. VT-PXRD of the **1**_0.46_**2**_0.54_ liquid showed a scattering pattern at 95 °C in the cooling process, which also supports supercooling (Figure 2A). Peaks from **1** and **2** at 80 °C in the cooling process resulted from eutectic crystallization. We did not observe any peaks attributable to crystals other than **1** and **2**. The peaks from planes (1 0 1) of **2** (2θ = 13.3°), (1 0 3) of **2** (2θ = 19.2°), (2 0 2) of **1** (2θ = 27.0°), and (2 2 0) of **1** (2θ = 30.3°) did not reappear in the cooling process. The phenomenon is due to the result of the anisotropic crystal growth in the eutectic crystallization of **1**_0.46_**2**_0.54_. The anisotropic crystal growth macrostructure of solids from eutectic crystallization depends on processes of crystal nucleation and growth.^47^ We observed the lamellar macrostructure in **1**_0.46_**2**_0.54_ by polarized optical microscopy observation after the crystallization of the **1**_0.46_**2**_0.54_ liquid (Figure 2C). The lamellar structures were not observed in **1** and **2** liquids and crystallization (Figure S11). Lamellar macrostructures are common in binary compounds after solidifying liquids because the simultaneous crystallization of two constituents at *T*_e_ results in low diffusion of nuclei and slow crystal growth.^48,49^ Scanning electron microscopy (SEM) images of recrystallized **1**_0.46_**2**_0.54_ (Figure 2D) show the structure with striped textures of **1** and **2**, which also support the formation of a lamellar structure by eutectic crystallization.

DSC measurements with the scan rates of 20, 30, 75, and 100 °C min^–1^ showed endothermic peaks resulting from crystal melting at 100 °C with a Δ*H*_fus_ of 40 kJ mol^–1^ (Figure S12). We found exothermic peaks in the cooling rates of 20 and 30 °C min^–1^, but Δ*H*_c_ (27 and 13 kJ mol^–1^, respectively) values were smaller than that found in the cooling rate of 10 °C min^–1^ (40 kJ mol^–1^) due to low crystallinity. DSC measurements with scan rates of 75 and 100 °C min^–1^ did not show exothermic peaks in the cooling process, and the exothermic peaks resulting from cold crystallization appeared in the second and third heating processes. The results indicate that a 10 °C min^–1^ cooling process is required to reach equilibrium during the melting and crystallization processes.

We tracked the crystal melting and crystallization by VT-FT-IR measurements of **1**, **2**, and **1**_0.46_**2**_0.54_ (Figure S13). The CN stretching band (ν_CN_) of **1**_0.46_**2**_0.54_ (2274 cm^–1^ at 40 °C) shifted to 2272 cm^–1^ at 80 and 95 °C. The ν_CN_ of **1** and **2** also shifted to the lower-wavenumber side by heating. Cooling of **1**_0.46_**2**_0.54_, **1**, and **2** led to a higher-wavenumber side shift of the ν_CN_ due to crystallization. The lower-wavenumber shift arises from the increase of the electron density of an antibonding orbital of cyanides due to weak Ag–N coordination and long C≡N covalent bonds.^50^ Interfacial interaction leads to eutectic melting of binary compounds.^8^ The Ag^+^-dinitrile frameworks and labile Ag^+^-N coordination bonds facilitate the interfacial interaction between **1** and **2**, resulting in a eutectic melting.

### Binary Phase Diagrams of **3** and **4**

**Figure 3:**
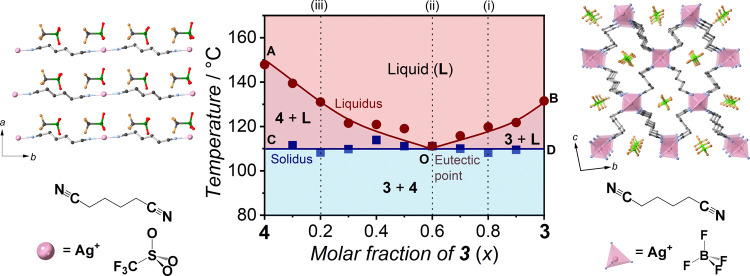
Binary phase diagram of Ag(AN)_2_(BF_4_) (**3**, right) and Ag(AN)(OTf) (**4**, left) with crystal structures and components of **3** and **4**. C, N, Ag, B, F, S, and O atoms are black, blue, pink, light green, orange, dark green, and red, respectively. Dashed lines marked as (i), (ii), and (iii) indicate the corresponding PXRD patterns shown in Figure S14.

Structural similarities of **1** and **2**, such as four-coordinated Ag^+^ ions in the tetrahedral geometries and 2D structures, lead to ligand exchange reactions that facilitate eutectic melting of **1**_*x*_**2**_1–*x*_. We examined other constituents that have less structural similarity in creating binary compounds. **3** has Ag^+^ ions in tetrahedral geometries and a three-dimensional (3D) Ag^+^-AN structure, while **4** has two-coordinated Ag^+^ ions and a one-dimensional (1D) Ag^+^-AN structure (Figure 3).^31^ Their structural difference arises from the different counteranions.

PXRD patterns of **3**_*x*_**4**_1–*x*_ (*x* = 0.1, 0.2, 0.3, 0.4, 0.5, 0.6, 0.7, 0.8, and 0.9) consist of peaks derived from **3** and **4**, suggesting that no chemical reaction and amorphization occurred in the mechanical mixing process (Figure S6B). *T*_e_ was observed at 110 °C in the DSC conducted for **3**_*x*_**4**_1–*x*_ (*x* = 0.1, 0.2, 0.3, 0.4, 0.5, 0.6, 0.7, 0.8, and 0.9, Figure S7B). **3**_0.6_**4**_0.4_, having only one *T*_m_ at *T*_e_, is in the eutectic composition (Table S6). Figure 3 shows the binary phase diagram of **3**–**4**. Phases described in the diagrams are determined by the VT-PXRD of **3**_0.8_**4**_0.2_, **3**_0.6_**4**_0.4_, and **3**_0.2_**4**_0.8_ (compositions shown as (i), (ii), and (iii) in Figure 3, Figure S14). The diagram represents a binary eutectic system without a terminal solid solution. The two-coordinated Ag^+^ ions in **4** have the capability to accept more ligands and facilitate ligand exchange reactions between **3** and **4** in an associative substitution mechanism, resulting in the eutectic melting, like the **1**–**2** system.

### Binary Phase Diagrams of **3** and **5** Showing the Terminal Solid Solution Phase

Binary metals have a terminal solid solution phase when the metals have similar atomic sizes, valences, and crystal space groups according to the Hume–Rothery rules.^51^ CPs also follow the rules when two crystal structures are similar (i.e., coordination geometry of metal ions, dimension of structure).^52^ We examined **3** and **5** having structural similarity of Ag^+^ ions in tetrahedral geometries and 3D Ag^+^-AN structures in creating binary compounds.^31^**5** accommodates PF_6_^–^ (thermochemical radii: 2.42 Å) and **3** accommodates BF_4_^–^ (thermochemical radii: 2.05 Å), resulting in different lattice dimensions.^53^

**Figure 4:**
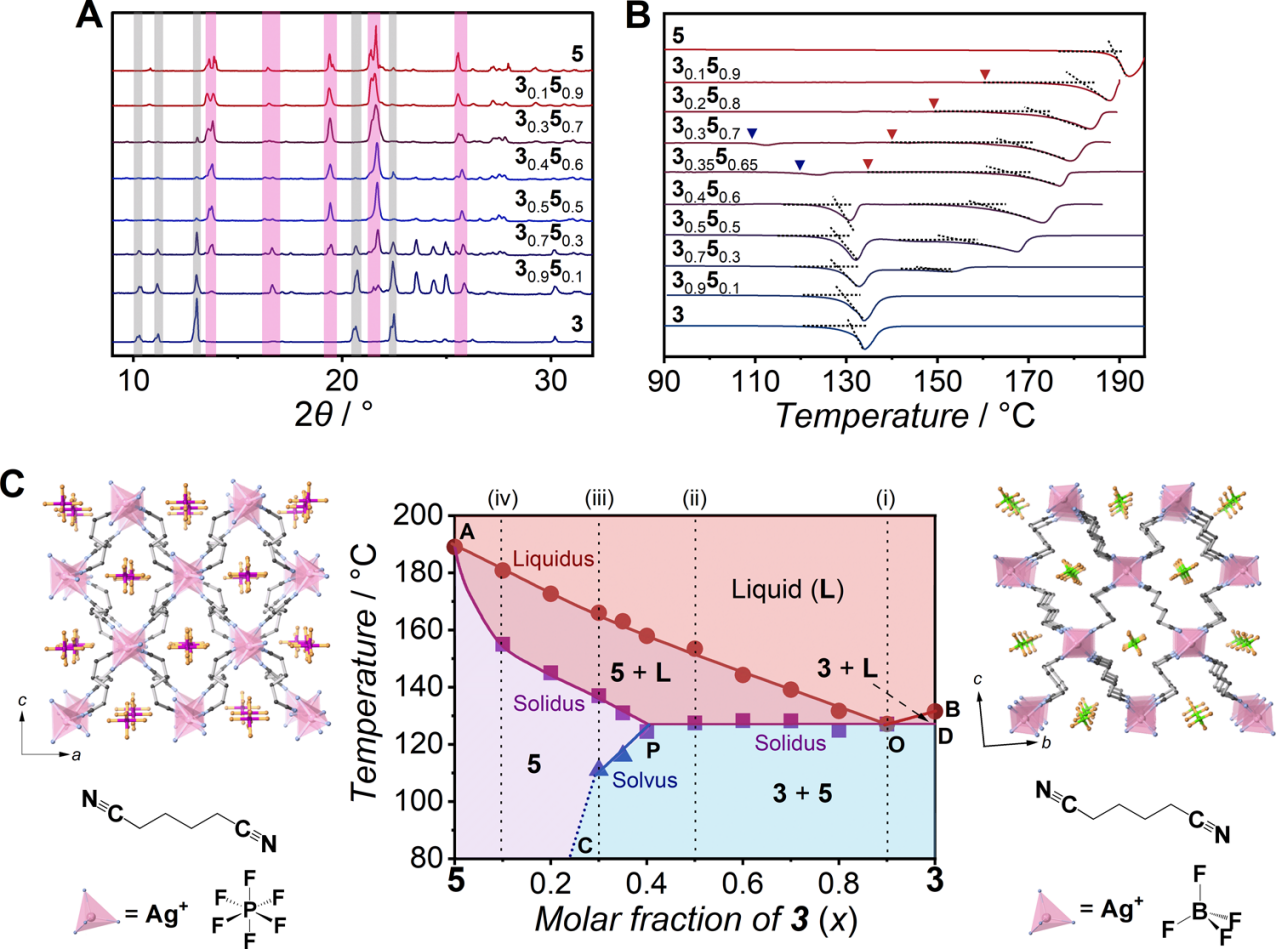
(A) PXRD patterns of **3**_*x*_**5**_1–*x*_ at room temperature. Color masks represent diffraction peaks derived from **3** (gray) and **5** (pink), respectively. (B) DSC curves of **3**_*x*_**5**_1–*x*_ in the upscan. The inset points (red triangles) representing the solidus of solid solutions are defined as the first deviation from baselines for **3**_0.35_**5**_0.65_, **3**_0.3_**5**_0.7_, **3**_0.2_**5**_0.8_, and **3**_0.1_**5**_0.9_. Endothermic peaks of the solvus are shown by blue triangles for **3**_0.3_**5**_0.7_ and **3**_0.35_**5**_0.65_. (C) Binary phase diagram of **3**_*x*_**5**_1–*x*_ with crystal structures and components of **3** (right) and **5** (left). C, N, Ag, B, F, O, and P atoms are black, blue, pink, green, orange, red, and purple, respectively. Dashed lines marked as (i), (ii), (iii), and (iv) indicate the corresponding PXRD patterns shown in Figure S15.

PXRD patterns of **3**_*x*_**5**_1–*x*_ in **3**-rich compositions (*x* = 0.3, 0.4, 0.5, 0.7, and 0.9) consist of peaks derived from **3** and **5** at room temperature, suggesting that no chemical reaction and amorphization occurred in the mechanical mixing process (Figure 4A). *T*_e_ was found at 127 °C in DSC conducted for **3**_*x*_**5**_1–*x*_ (*x* = 0.4, 0.5, 0.6, 0.7, 0.8, and 0.9, Figure 4B and Table S7). **3**_0.9_**5**_0.1_ has only one *T*_m_ at *T*_e_ and showed a scattered PXRD pattern at 130 °C, which is higher than *T*_e_ (Figure S15A). **3**_*x*_**5**_1–*x*_ (*x* = 0.4, 0.5, 0.6, 0.7, and 0.8) has the equilibrium *T*_m_ at 132, 139, 144, 153, and 158 °C, respectively. The VT-PXRD of **3**_0.5_**5**_0.5_ indicates the successive melting of **3** and **5** at *T*_e_ (127 °C by DSC) and the equilibrium *T*_m_ (153 °C by DSC), respectively (Figure S15B). The DSC curves of **3**_0.3_**5**_0.7_ and **3**_0.35_**5**_0.65_ have broad endothermic peaks above 160 °C, which are characteristic of solid solutions (Figure 4B). The inset and onset temperatures represent the solidus and liquidus, respectively.^39,40^ Their crystal melting is characterized by scattering PXRD patterns at 180 °C (Figure S15C). **3**_0.3_**5**_0.7_ and **3**_0.35_**5**_0.65_ have small endothermic peaks at 111 and 116 °C, respectively, below *T*_e_ (127 °C). The peaks indicate the solvus where **3** completely dissolves in **5**. PXRD patterns of **3**_0.3_**5**_0.7_ consist of peaks derived from **3** and **5** at 40, 60, and 100 °C (Figures 4A and S15C). The disappearance of **3** peaks at 120, 140, and 160 °C also indicates that **3** completely dissolves in **5** at the solvus. The DSC curves of **3**_0.1_**5**_0.9_ and **3**_0.2_**5**_0.8_ have broad endothermic peaks representing the solidus and liquidus but no peaks representing the solvus. PXRD patterns of **3**_0.2_**5**_0.8_ have no peaks arising from **3** at 40, 60, 120, and 150 °C because **3**_0.2_**5**_0.8_ is below the maximum solubility limit of **3** in **5** (Figure S15D).

PXRD patterns of **3**_0.3_**5**_0.7_ consist of peaks derived from **3** and **5** at 40, 60, and 100 °C (Figures 4A and S15C). The disappearance of **3** peaks at 120, 140, and 160 °C indicates that **3** completely dissolves in **5** at the solvus. The DSC curves of **3**_0.1_**5**_0.9_ and **3**_0.2_**5**_0.8_ have broad endothermic peaks representing the solidus and liquidus but no peaks representing the solvus. PXRD patterns of **3**_0.2_**5**_0.8_ have no peaks arising from **3** at 40, 60, 120, and 150 °C. **3**_0.2_**5**_0.8_ is below the maximum solubility limit of **3** in **5** (Figure S15D).

We depicted a composition–temperature binary phase diagram of the **3**–**5** system showing a binary eutectic system with a terminal solid solution, like the Sn–Pb system (Figure 4C).^41^ The diagram consists of two liquidus lines (AO and BO), two solidus lines (AP and DP), and a solvus line (CP). Part of the solvus line is shown as a dotted line because of uncertainties around the maximum solubility limit as observed in binary metals.^8^

We analyzed the solid solution structure using the pair distribution function (PDF) analysis from X-ray total scattering (Figure S16). **3**_0.2_**5**_0.8_ does not have peaks contributed by the distances of neighboring Ag^+^ ions in **3** (7.7 and 11.6 Å) but shows peaks at 6.5 and 12.6 Å dominated by the distances of neighboring Ag^+^ ions in **5**. The SEM image of **3**_0.2_**5**_0.8_ with elemental mapping indicates the coexistence of BF_4_^–^ and PF_6_^–^ in **3**_0.2_**5**_0.8_ particles (Figure S17). Diffraction peaks of (200), (020), and (002) found at 2θ = 16.49, 13.68, and 13.91°, respectively, for **5**, shifted to 2θ = 16.48, 13.53, and 13.82°, respectively, for **3**_0.1_**5**_0.9_ and **3**_0.3_**5**_0.7_. The difference of lattice constants of **5** (*a* = 10.738 Å, *b* = 12.931 Å, and *c* = 12.719 Å), and **3**_0.1_**5**_0.9_ and **3**_0.3_**5**_0.7_ (*a* = 10.745 Å, *b* = 13.073 Å, and *c* = 12.800 Å) results from the unit cell expansion by the anion exchange of PF_6_^–^ with BF_4_^–^. ν_CN_, ν_PF_, and ν_BF_ peaks were observed in the FT-IR spectra of **3**, **5**, and **3**_*x*_**5**_1–x_ (*x* = 0.1, 0.2, 0.3, 0.5, 0.7, 0.8, and 0.9), but specific peak shifts arising from BF_4_^–^ in the **5** frameworks were not found in **3**_0.1_**5**_0.9_ and **3**_0.3_**5**_0.7_ (Figure S18).

### Latent Heat Storage Functions of Binary Compounds

Reversible solid–liquid phase transitions are applicable to latent heat storage (LHS).^54,55^ LHS materials store and release thermal energy at *T*_m_ and *T*_c_, respectively, and should have a large heat storage capacity estimated from Δ*H*_fus_ and crystallization enthalpy (Δ*H*_c_) and high energy recovery efficiency (Δ*H*_s_/Δ*H*_fus_ ∼ 1).^56^ LHS materials with large phase-transition hysteresis defined by *T*_m_–*T*_c_ are beneficial to long-term heat storage with minimum insulation and stimuli-responsible heat release.^57^ To date, about 20 binary compounds are in practical use. Binary organic materials such as erythritol-urea mostly work below 80 °C, and inorganic materials such as NaNO_3_–KNO_3_ work above 200 °C.^58−,60^ Sugar alcohols are representative LHS materials available at around 100 °C. Erythritol melts at 119 °C, crystallizes at 22 °C, and has an energy recovery efficiency of 56%.^100^ The large hysteresis is beneficial but the energy recovery efficiency is low due to formation of an amorphous solid.

**1** stores and releases heat at 147 and 143 °C, respectively, and has a heat storage capacity of 112 kJ kg^–1^, which is in the range of practical organic materials (Table S4, 90–248 kJ kg^–1^). The energy recovery efficiency of **1** (0.98) is as high as that of [Ca(*N*-methylurea)_6_](NO_3_)_2_ (0.98), which has the highest efficiency among the reported CP/MOFs showing LHS.^17^ The high efficiency is due to the absence of amorphization and cold crystallization typically observed in other LHS materials. **1** is promising as an intermediate-temperature LHS but the phase-transition hysteresis window is 4 °C, which is not sufficient for long-term heat storage. **2** shows similar LHS properties as shown in Table S4. The binary systems have wider phase-transition hysteresis because of the supercooling state resulting from low diffusion of nuclei and slow crystal growth in the binary liquid.^57^ We investigated the binary phase diagrams of **1** and **2** to explore optimal LHS properties and found that **1**_0.46_**2**_0.54_ shows a heat storage capacity of 97 kJ kg^–1^ (Table S8). It stores and releases heat at 100 and 80 °C, respectively, and the phase-transition hysteresis window is 20 °C, which is four times larger than those of **1** and **2**. The hysteresis window is comparable to poly(ethylene glycol) 4000 (22 °C), recognized as an LHS material with large hysteresis.^61^ The energy recovery efficiency of **1**_0.46_**2**_0.54_ is 0.99, which results from eutectic crystallization without solid solution formation in the **1**–**2** system.

## Conclusions

We demonstrated the construction of three types of binary phase diagrams using Ag^+^-based coordination polymer (CP) crystals. Based on the five compounds from **1** to **5** with different compositions and structure dimensionalities, we prepared the binary systems and investigated their structural/thermal properties. All diagrams showed lower *T*_m_ values than the single components due to the eutectic phenomenon. The binary system **3**–**5** only showed terminal solid solution phases, which were determined by the matching of crystal structures, coordination geometries, and compositions. X-ray diffraction and elemental mapping suggested that solid solution formation is promoted by both ligands and anion exchange. Crystallization of the binary liquids on cooling resulted in the formation of a lamellar macrostructure and supercooling due to low nuclear diffusion and slow crystal growth. The optimal binary compound **1**_0.46_**2**_0.54_ in the eutectic ratio represented the heat storage capacity of 97 kJ kg^–1^, the energy recovery efficiency of 0.99, and phase-transition hysteresis (20 °C) to store and release heat at 100 and 80 °C, respectively. The properties are promising for reuse of waste heat from industries at intermediate-temperature ranges, which is not accessible using conventional organic and inorganic LHS materials. CP/MOFs consisting of dinitrile, *N*-methylurea, and acetamide ligands exhibiting reversible melting and recrystallization are available as constituents of binary systems.^17^ The investigation of a binary phase diagram of these CP/MOFs will be a powerful approach to exploring the unknown phases and functionalities.

## Supporting Information Available

PXRD patterns, FT-IR spectra, TG-DTA profiles, DSC profiles, modeled binary phase diagram, SEM and elemental mapping images, PDF profiles, tables of thermophysical parameters, and crystallographic parameters of **5** (PDF)
