# Supplementary material for: Binary Phase Diagrams of Coordination Polymers with Eutectic Behaviors
Source: J Am Chem Soc. 2025 Feb 3;147(6):5140–8. doi: 10.1021/jacs.4c15317 (PMC11826988; doi:10.1021/jacs.4c15317)
Supplement: Supplementary file 1 — ja4c15317_si_001.pdf [file ja4c15317_si_001.pdf]

*Supporting Information for*

# Binary Phase Diagrams of Coordination Polymers with Eutectic Behaviors

Karnjana Atthawilai,[a] Hiroyasu Tabe,*[b] Kotaro Ohara,[c] Kanokwan Kongpatpanich,[a] Satoshi Horike*[abd]

[a] Department of Materials Science and Engineering, School of Molecular Science and Engineering, Vidyasirimedhi Institute of Science and Technology, Rayong 21210, Thailand

[b] Institute for Integrated Cell-Material Sciences, Institute for Advanced Study, Kyoto University, Yoshida-Hommachi, Sakyo-ku, Kyoto 606-8501, Japan

[c] Department of Synthetic Chemistry and Biological Chemistry, Graduate School of Engineering, Kyoto University, Katsura, Nishikyo-ku, Kyoto 615-8510, Japan

[d] Department of Chemistry, Graduate School of Science, Kyoto University, Kitashirakawa-Oiwakecho, Sakyo-ku, Kyoto 606-8502, Japan

*E-mail: tabe.hiroyasu.4y@kyoto-u.ac.jp

*E-mail: horike.satoshi.3r@kyoto-u.ac.jp

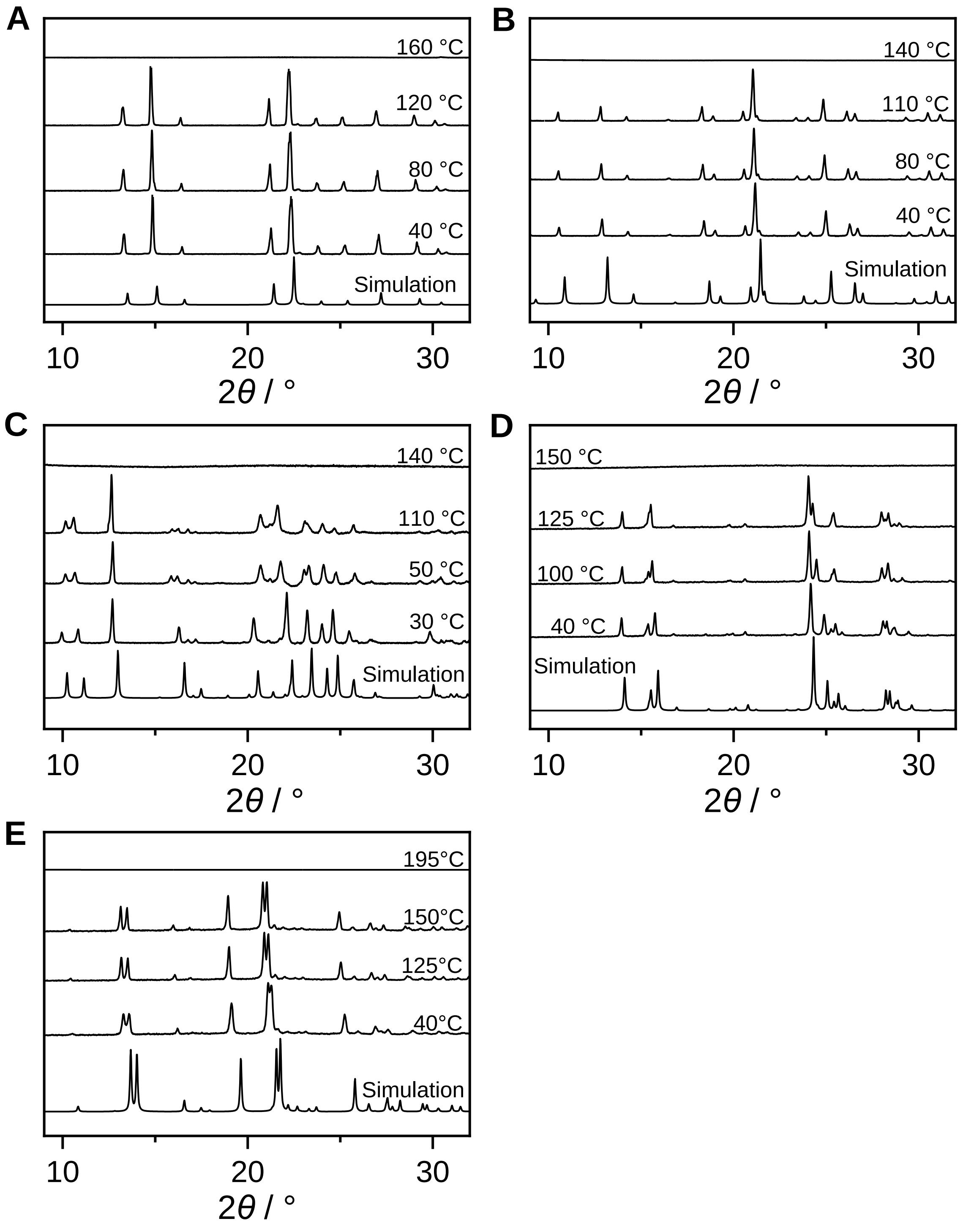


**Figure S1.** Powder X-ray diffraction (PXRD) patterns of (A) $Ag(GN)_2(BF_4)$ (**1**), (B) $Ag(PN)_2(BF_4)$ (**2**), (C) $Ag(AN)_2(BF_4)$ (**3**), (D) Ag(AN)(OTf) (**4**), and (E) $Ag(AN)_2(PF_6)$ (**5**) at variable temperatures.

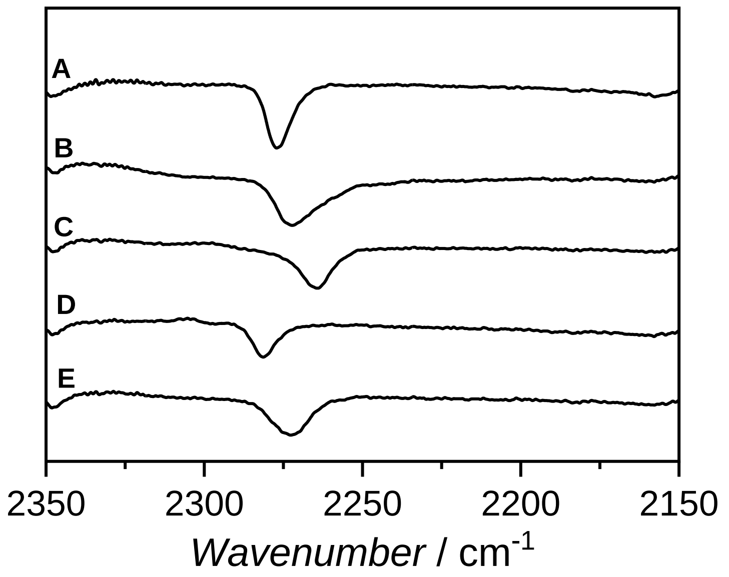


**Figure S2.** CN-stretching ($\nu_{CN}$) region of FT-IR spectra of (A) $Ag(GN)_2(BF_4)$ (**1**), (B) $Ag(PN)_2(BF_4)$ (**2**), (C) $Ag(AN)_2(BF_4)$ (**3**), (D) Ag(AN)(OTf) (**4**), and (E) $Ag(AN)_2(PF_6)$ (**5**).

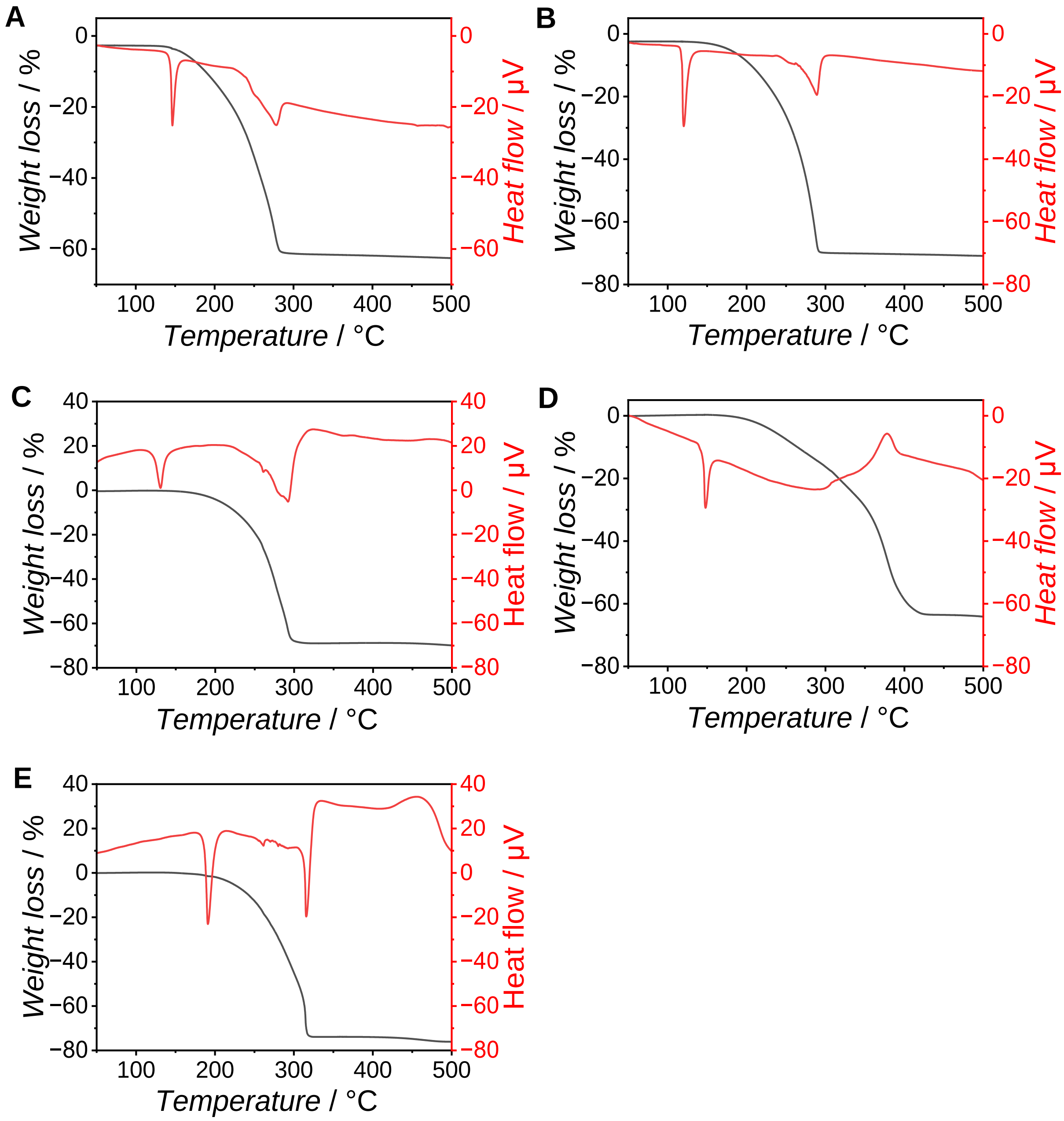


**Figure S3.** Thermogravimetric analyses (TGA) and differential thermal analysis (DTA) profiles of (A) $Ag(GN)_2(BF_4)$ (**1**), (B) $Ag(PN)_2(BF_4)$ (**2**), (C) $Ag(AN)_2(BF_4)$ (**3**), (D) Ag(AN)(OTf) (**4**), and (E) $Ag(AN)_2(PF_6)$ (**5**).

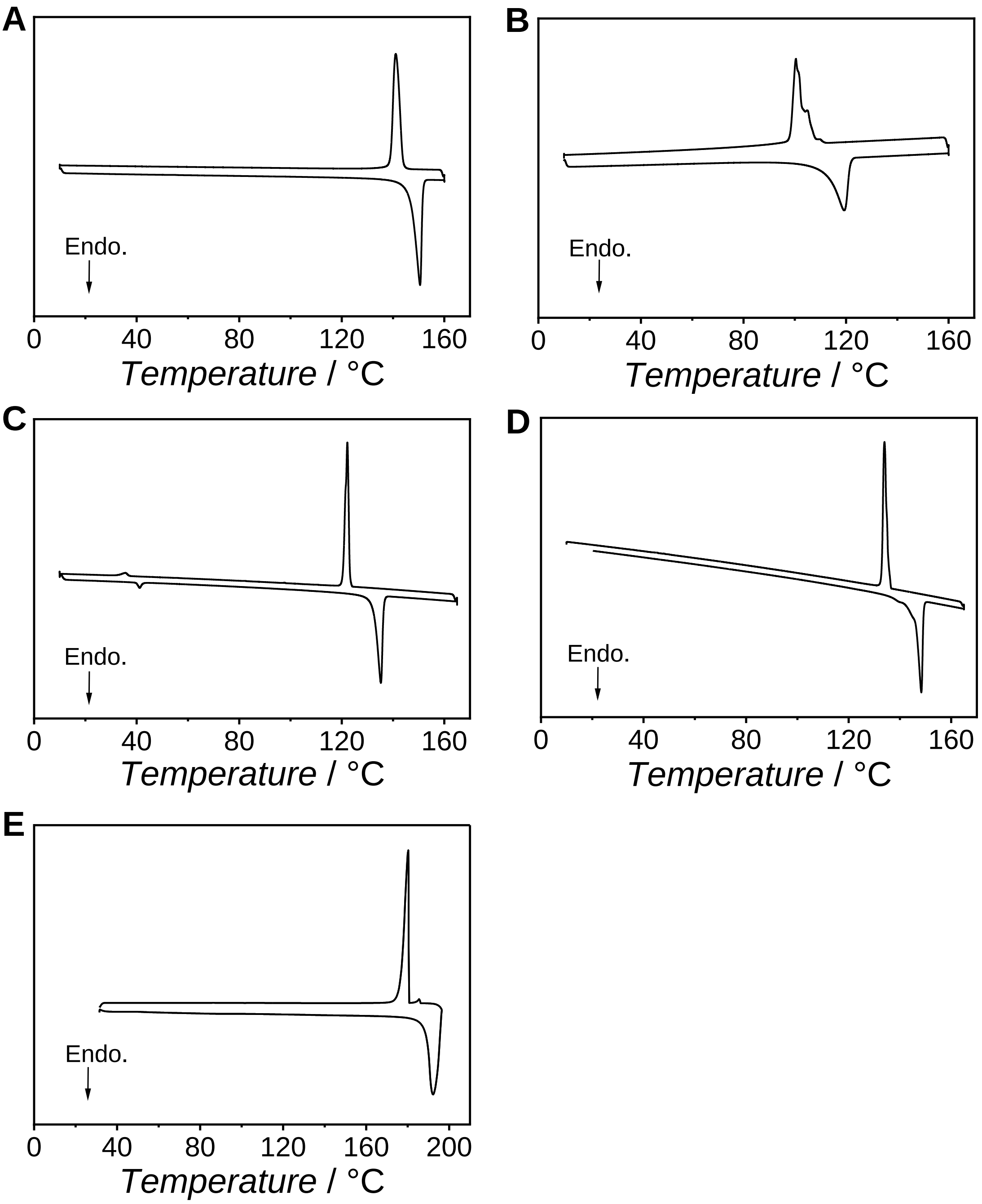


**Figure S4.** Differential scanning calorimetry (DSC) profiles of (A) $Ag(GN)_2(BF_4)$ (**1**), (B) $Ag(PN)_2(BF_4)$ (**2**), (C) $Ag(AN)_2(BF_4)$ (**3**), (D) Ag(AN)(OTf) (**4**), and (E) $Ag(AN)_2(PF_6)$ (**5**).

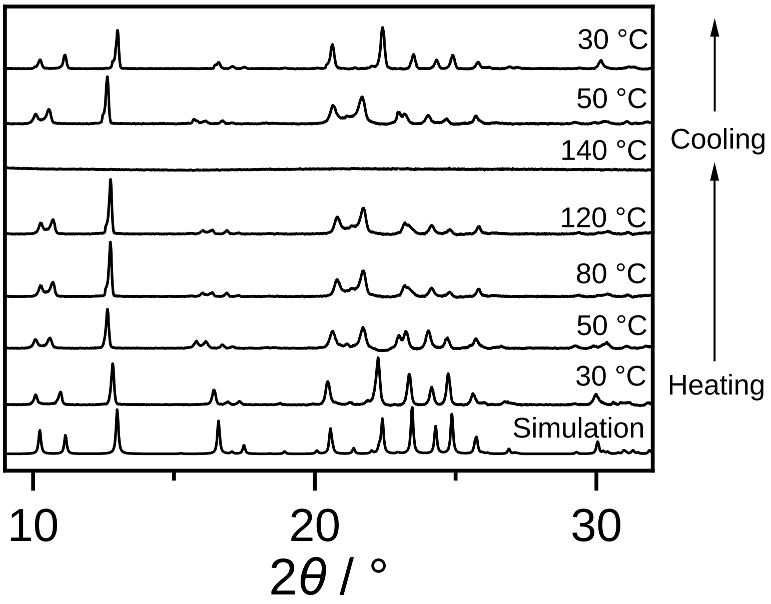


**Figure S5.** Powder X-ray diffraction (PXRD) patterns of $Ag(AN)_2(BF_4)$ (**3**) at variable temperatures in the heating and cooling processes.

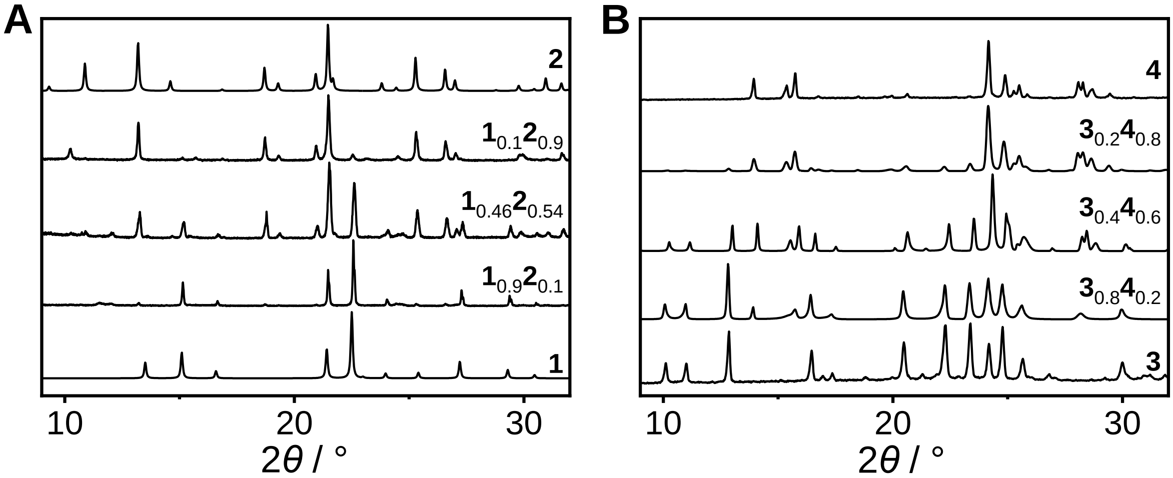


**Figure S6.** PXRD patterns of (A) $\mathbf{1}_x\mathbf{2}_{1-x}$ and (B) $\mathbf{3}_x\mathbf{4}_{1-x}$ at room temperature.

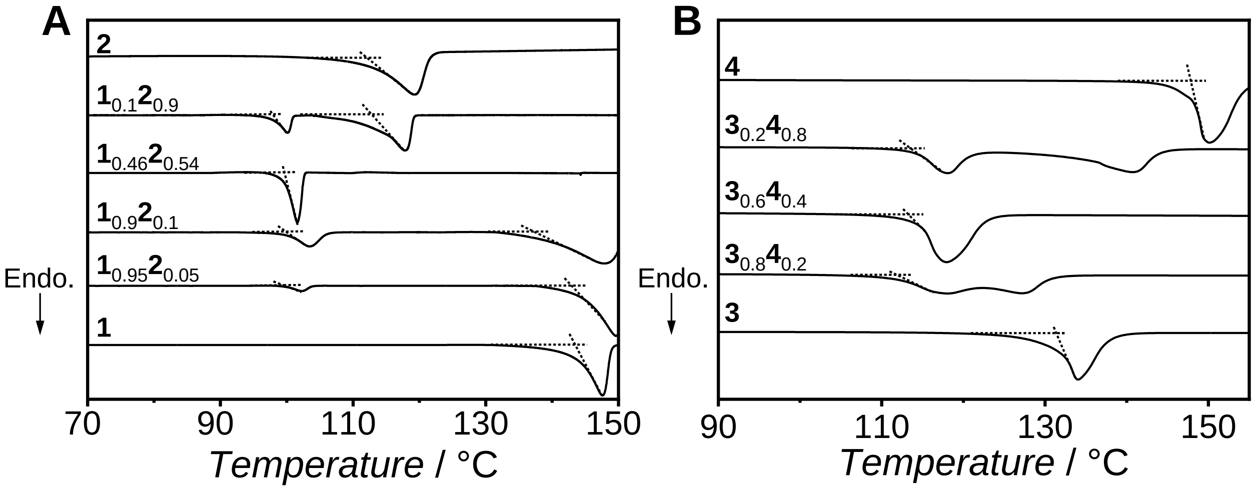


**Figure S7.** DSC profiles of (A) $\mathbf{1}_x\mathbf{2}_{1-x}$ and (B) $\mathbf{3}_x\mathbf{4}_{1-x}$.

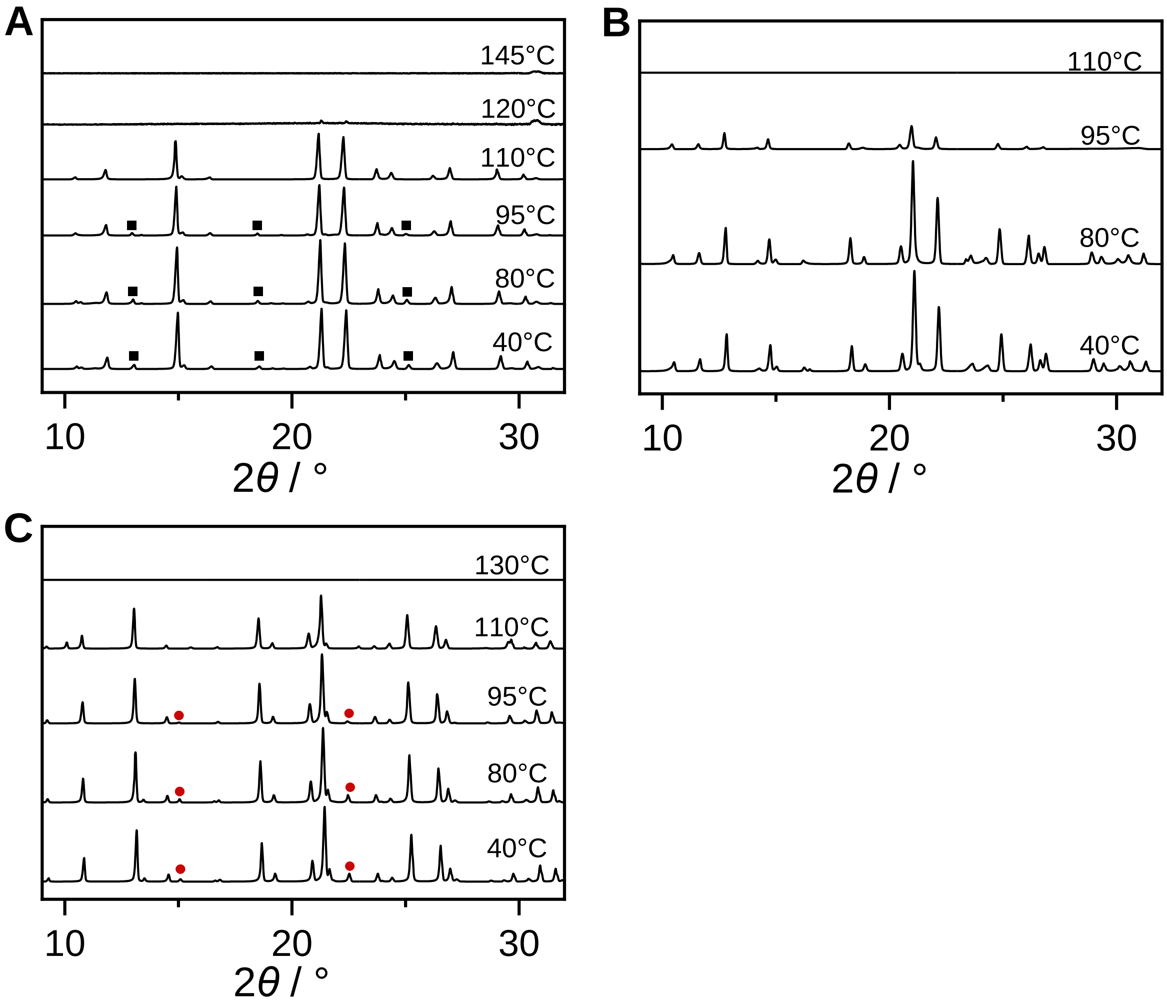


**Figure S8.** PXRD patterns of (A) $\mathbf{1}_{0.9}\mathbf{2}_{0.1}$, (B) $\mathbf{1}_{0.46}\mathbf{2}_{0.54}$, and (B) $\mathbf{1}_{0.1}\mathbf{2}_{0.9}$ at variable temperatures. Several peak positions of **1** and **2** overlapped. ■ in (A) and ● in (C) represent the diffraction peaks derived from **2** and **1**, respectively.

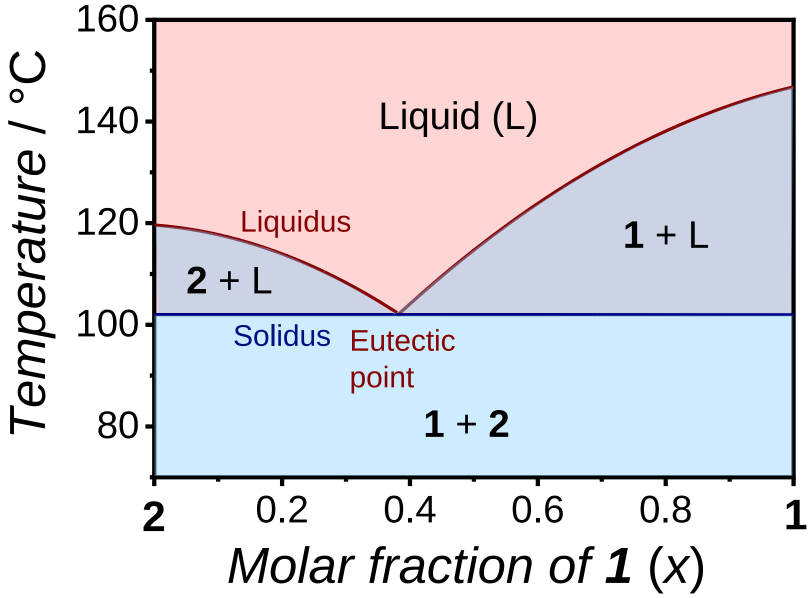


**Figure S9.** Binary phase diagram of **1** and **2** estimated from Schroeder-van Laar's equation.

**Note**: The equation shows the estimated eutectic ratio and $T_e$ in ideal binary systems. The interaction parameter ($\chi$) should be involved in the equation for non-ideal systems. We estimated $\chi$ as −1.28 using $T_{mi}$ and $\Delta H_{fusi}$ ($i$ = **1** and **2**) according to eqs. (1) and (2) where $R$ is the universal gas constant.[S1] We then determined the eutectic composition and $T_e$ as the intersection of the two branches of estimated liquidus lines.

$$\ln \gamma_i = \chi (1 - x_i)^2 \qquad \cdots \text{ eq. (1)}$$

$$\ln \gamma_i x_i = \frac{\Delta H_{fusi}}{R}\left(\frac{1}{T_{mi}} - \frac{1}{T}\right) \qquad \ldots \text{ eq. (2)}$$

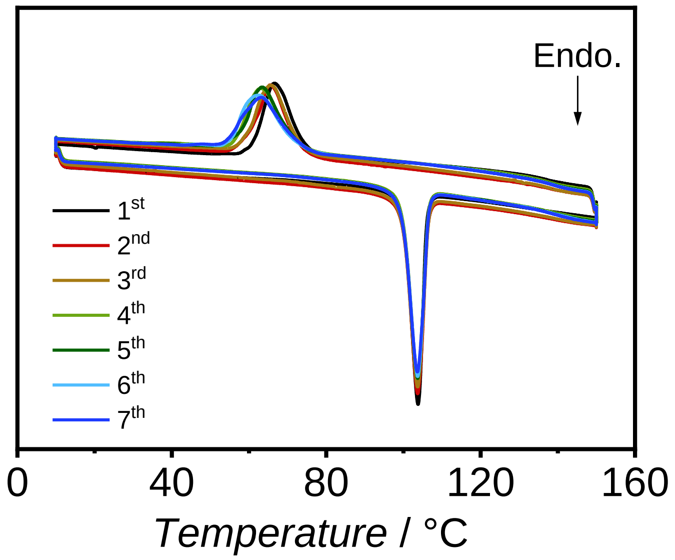


**Figure 10.** DSC profiles of $\mathbf{1}_{0.46}\mathbf{2}_{0.54}$ in seven heating-cooling cycles. 1st cycle: black, 2nd cycle: red, 3rd cycle: orange, 4th cycle: light green, 5th cycle: green, 6th cycle: light blue, 7th cycle: blue.

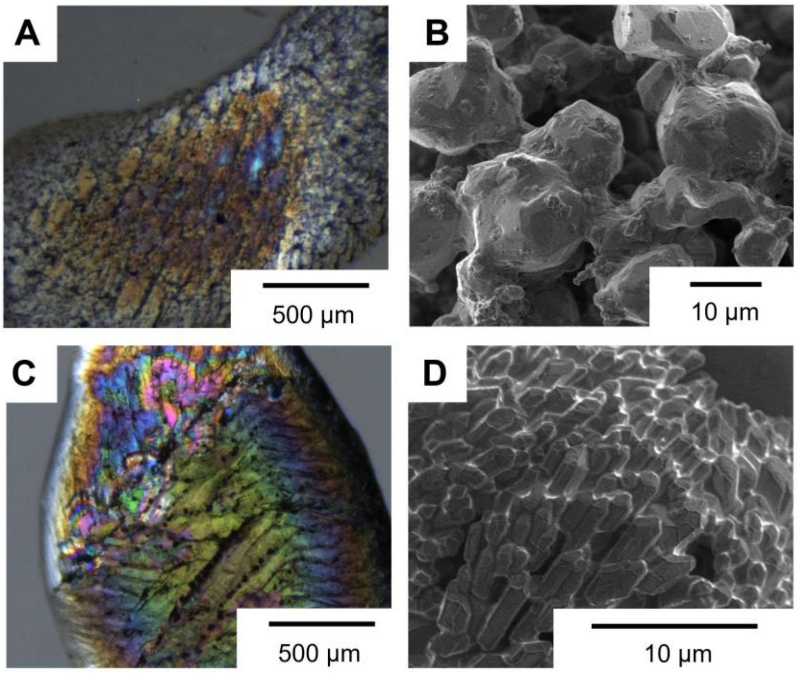


**Figure S11.** (A) Optical microscope and (B) SEM images of **1** after crystallization. (C) Optical microscope and (D) SEM images of **2** after crystallization.

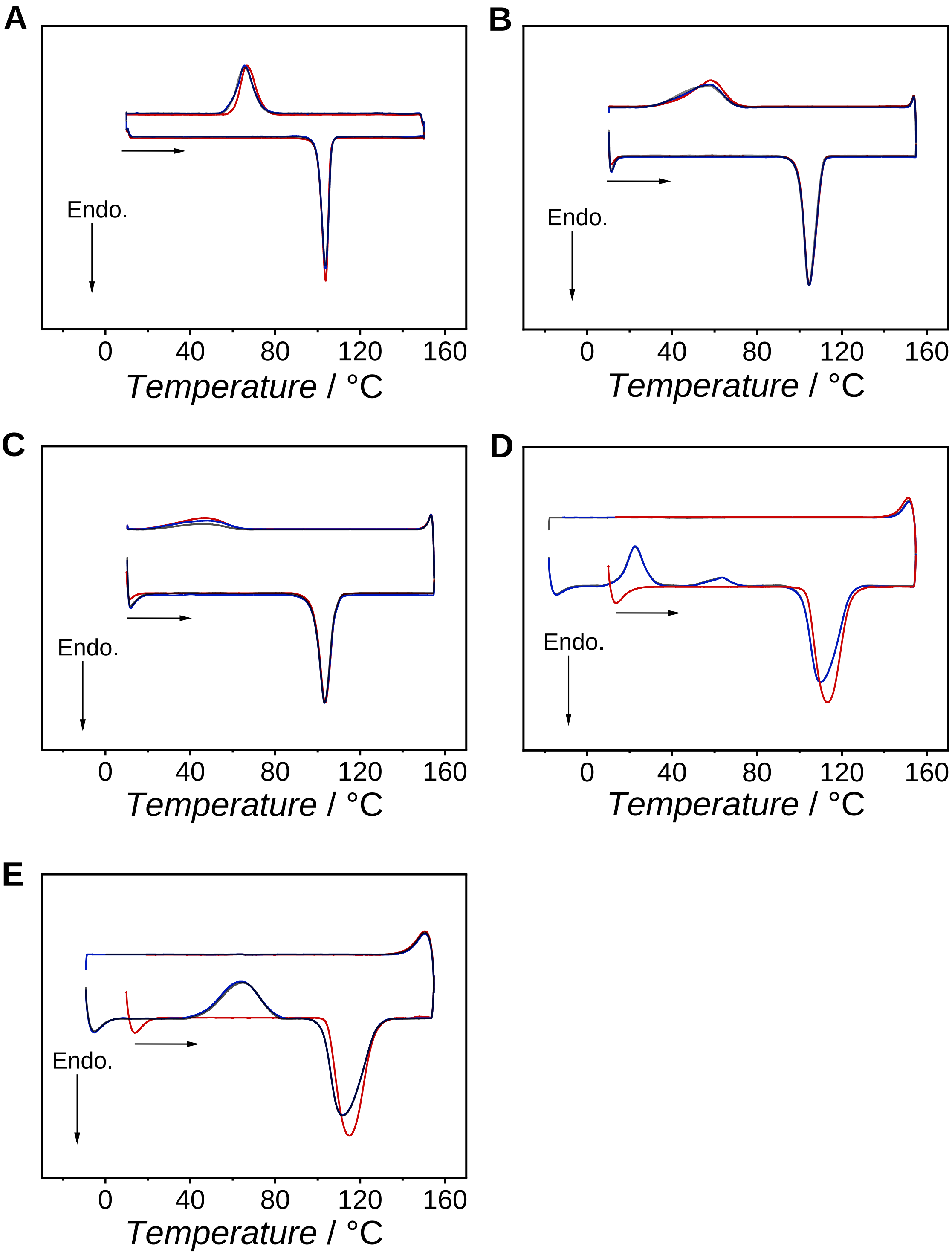


**Figure S12.** DSC profiles of $\mathbf{1}_{0.46}\mathbf{2}_{0.54}$ with scan rates of (A) 10, (B) 20, (C) 30, (D) 75, and (E) 100 °C min$^{-1}$. Red, blue, and black lines represent the first, second, and third scans, respectively.

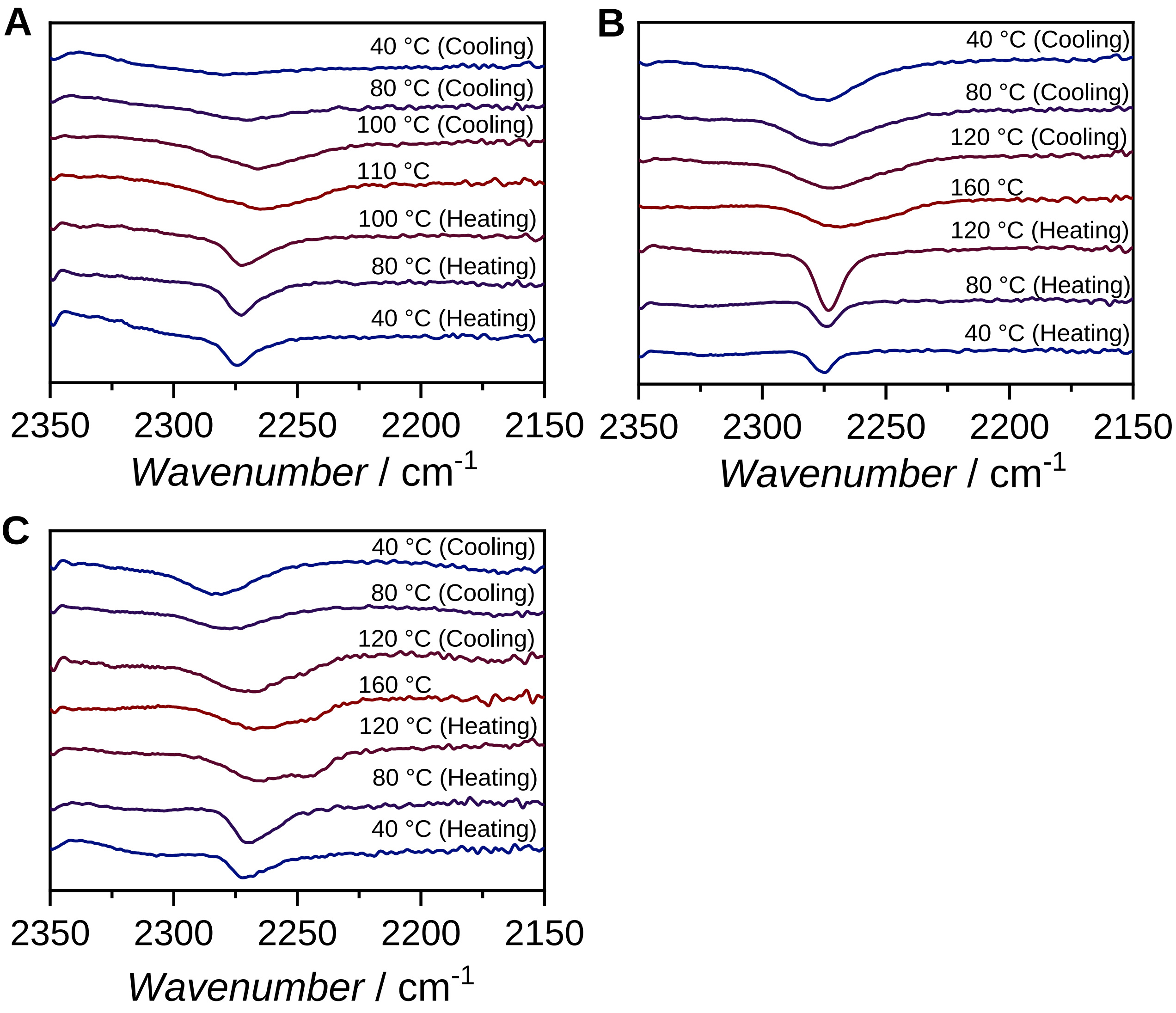


**Figure S13.** IR spectra of (A) $\mathbf{1}_{0.46}\mathbf{2}_{0.54}$, (B) **1**, and (C) **2** at various temperatures in the heating and cooling processes.

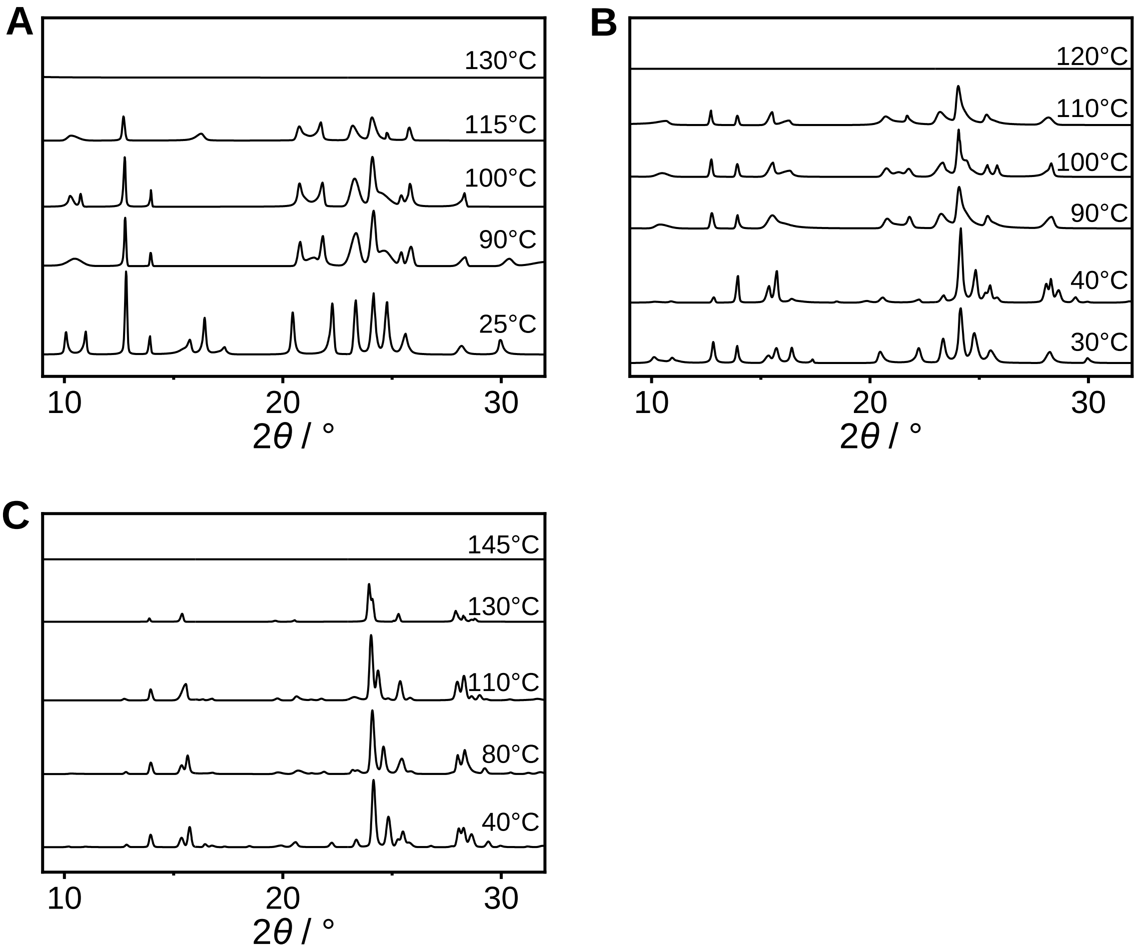


**Figure S14.** PXRD patterns of (A) $\mathbf{3}_{0.8}\mathbf{4}_{0.2}$, (B) $\mathbf{3}_{0.6}\mathbf{4}_{0.4}$, and (C) $\mathbf{3}_{0.2}\mathbf{4}_{0.8}$ at variable temperatures in the heating process.

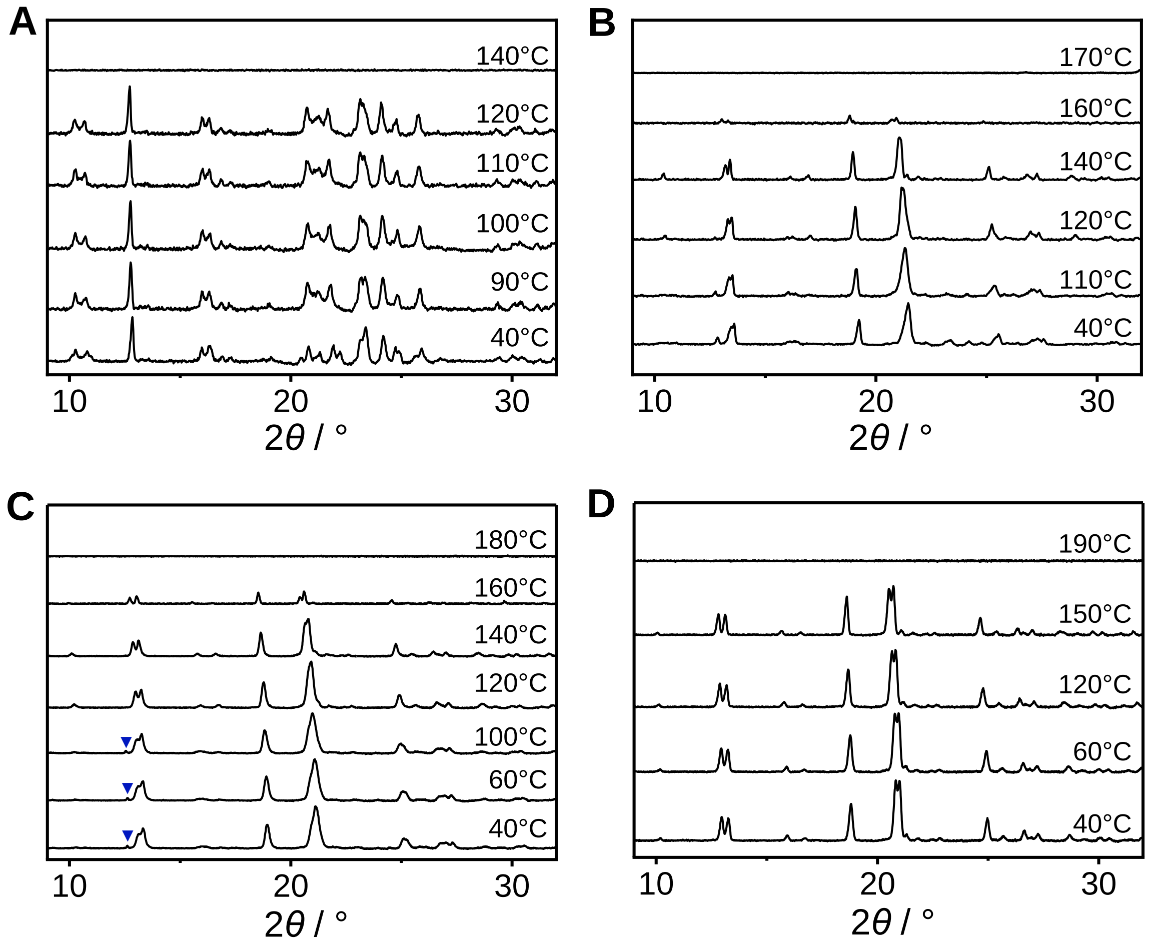


**Figure S15.** PXRD patterns of (A) $\mathbf{3}_{0.9}\mathbf{5}_{0.1}$, (B) $\mathbf{3}_{0.5}\mathbf{5}_{0.5}$, and (C) $\mathbf{3}_{0.3}\mathbf{5}_{0.7}$, and (D) $\mathbf{3}_{0.1}\mathbf{5}_{0.9}$ at variable temperatures in the heating process. ▼ in (C) indicate the peaks derived from **3**.

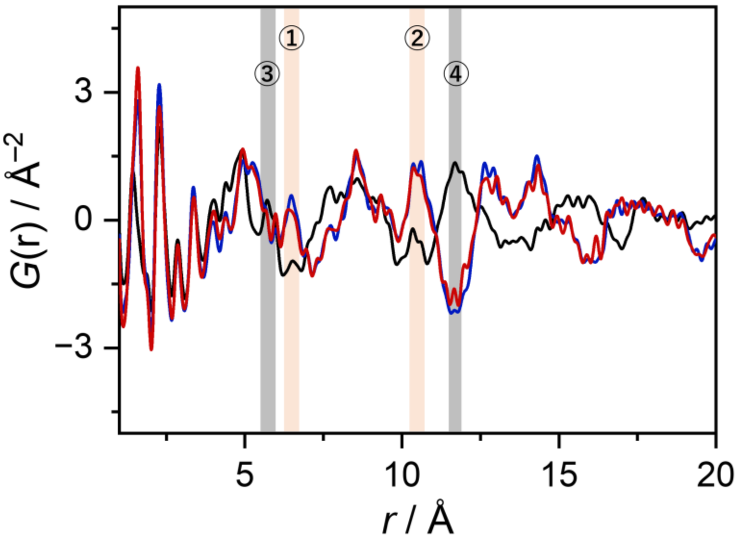


**Figure S16.** Pair distribution function (PDF) profiles from X-ray total scattering of **3** (black), **5** (blue), and $\mathbf{3}_{0.2}\mathbf{5}_{0.8}$ (red). ① and ② peaks represent Ag-Ag distances in **5**, and ③ and ④ peaks represent Ag-Ag distances in **3**.

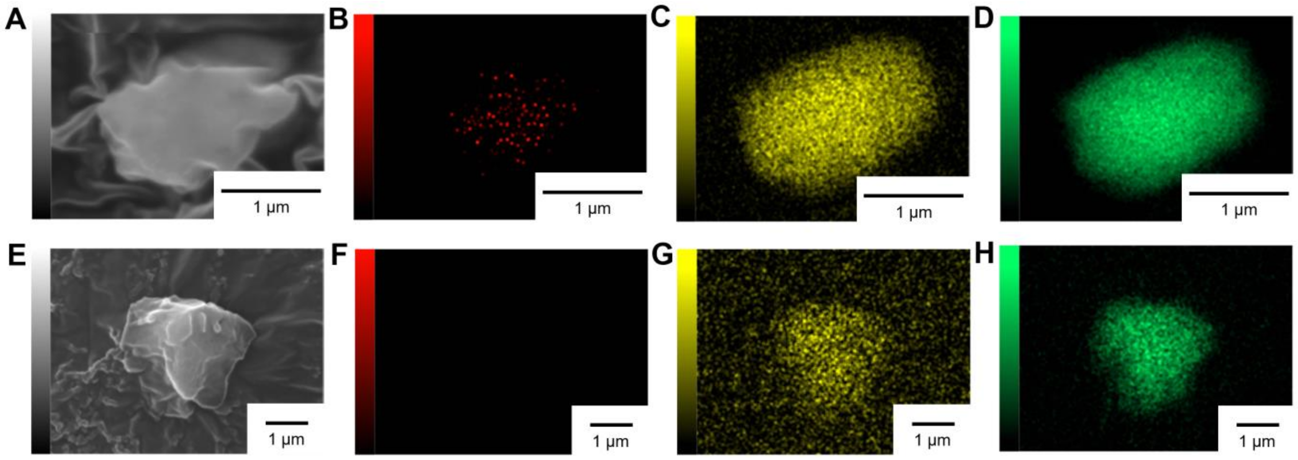


**Figure S17.** SEM images and elemental distributions obtained by energy dispersive X-ray spectroscopy (EDS) of $\mathbf{3}_{0.2}\mathbf{5}_{0.8}$ and **5**. (A) SEM image of $\mathbf{3}_{0.2}\mathbf{5}_{0.8}$. (B) B, (C) Ag, and (D) P atom distributions in $\mathbf{3}_{0.2}\mathbf{5}_{0.8}$. (E) SEM image of **5**. (F) B, (G) Ag, and (H) P atom distributions in **5**.

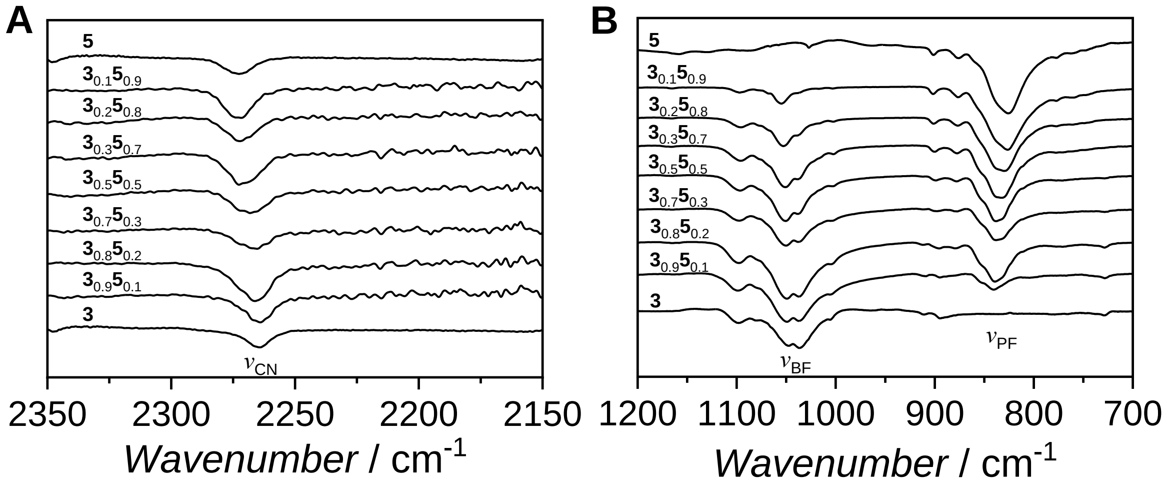


**Figure S18.** (A) CN-stretching ($\nu_{CN}$) region, (B) BF-stretching ($\nu_{BF}$) and PF-stretching ($\nu_{PF}$) regions of FT-IR spectra of Ag(AN)$_2$(BF$_4$) (**3**), Ag(AN)$_2$(PF$_6$) (**5**), and $\mathbf{3}_x\mathbf{5}_{1-x}$.

**Table S1.** Melting ($T_m$), crystallization ($T_c$), and decomposition ($T_d$) temperatures for each compound from DSC and TG/DTA.

| No. | Formula | $T_m$ / °C | $T_c$ / °C | $T_d$ / °C |
|---|---|---|---|---|
| **1** | $Ag(GN)_2(BF_4)$ | 147 | 143 | 161 |
| **2** | $Ag(PN)_2(BF_4)$ | 120 | 115 | 156 |
| **3** | $Ag(AN)_2(BF_4)$ | 132 | 128 | 150 |
| **4** | Ag(AN)(OTf) | 148 | 134 | 173 |
| **5** | $Ag(AN)_2(PF_6)$ | 189 | 180 | 213 |

**Table S2.** Crystallographic parameters of **5**.

| | |
|---|---|
| Experimental formula | $AgC_{12}N_4H_{16}PF_6$ |
| Formula weight | 469.13 |
| Temperature / K | 293 |
| Crystal system | orthorhombic |
| Space group | *Pnna* |
| $Z$ | 4 |
| $a$ / Å | 10.6969(4) |
| $b$ / Å | 12.9598(5) |
| $c$ / Å | 12.6393(5) |
| $\alpha$ / ° | 90 |
| $\beta$ / ° | 90 |
| $\gamma$ / ° | 90 |
| $V$ / Å$^3$ | 1752.18(12) |
| Goodness-of-fit $F^2$ | 1.081 |
| $wR_2$ for all data | 0.1649(2508) |
| CCDC No. | 2286694 |

**Table S3.** Eutectic and equilibrium $T_m$ of $\mathbf{1}_x\mathbf{2}_{1-x}$.

| Sample | Eutectic $T_m$ | Equilibrium $T_m$ |
|---|---|---|
| **1** | – | 147 |
| $\mathbf{1}_{0.95}\mathbf{2}_{0.05}$ | 98 | 144 |
| $\mathbf{1}_{0.90}\mathbf{2}_{0.10}$ | 100 | 143 |
| $\mathbf{1}_{0.75}\mathbf{2}_{0.25}$ | 99 | 132 |
| $\mathbf{1}_{0.67}\mathbf{2}_{0.33}$ | 101 | 125 |
| $\mathbf{1}_{0.50}\mathbf{2}_{0.50}$ | 101 | 113 |
| $\mathbf{1}_{0.46}\mathbf{2}_{0.54}$ | 100 | 100 |
| $\mathbf{1}_{0.30}\mathbf{2}_{0.70}$ | 98 | 109 |
| $\mathbf{1}_{0.20}\mathbf{2}_{0.80}$ | 100 | 110 |
| $\mathbf{1}_{0.10}\mathbf{2}_{0.90}$ | 98 | 118 |
| $\mathbf{1}_{0.05}\mathbf{2}_{0.95}$ | 99 | 116 |
| **2** | – | 120 |

**Table S4.** Thermochemical parameters of **1**, $\mathbf{1}_{0.46}\mathbf{2}_{0.54}$, and **2**.

| | **1** | $\mathbf{1}_{0.46}\mathbf{2}_{0.54}$ | **2** |
|---|---|---|---|
| Melting temperature[a] ($T_m$) / ºC | 147 | 100 | 120 |
| Fusion enthalpy ($\Delta H_{fus}$) / kJ mol$^{-1}$ | 43 | 40 | 53 |
| Fusion enthalpy ($\Delta H_{fus}$) / kJ kg$^{-1}$ | 112 | 97 | 121 |
| Fusion entropy[b] ($\Delta S_{fus}$) / J mol$^{-1}$ K$^{-1}$ | 102 | 108 | 134 |
| Fusion entropy[b] ($\Delta S_{fus}$) / J kg$^{-1}$ K$^{-1}$ | 266 | 262 | 305 |
| Crystallization temperature ($T_c$) / ºC | 143 | 80 | 115 |
| Crystallization enthalpy ($\Delta H_c$) / kJ mol$^{-1}$ | 42 | 40 | 49 |
| Crystallization enthalpy ($\Delta H_c$) / kJ kg$^{-1}$ | 110 | 97 | 112 |
| Crystallization entropy ($\Delta S_c$) / J mol$^{-1}$ K$^{-1}$ | 100 | 112 | 126 |
| Crystallization entropy ($\Delta S_c$) / J Kg$^{-1}$ K$^{-1}$ | 261 | 271 | 287 |
| Hysteresis[c] / ºC | 4 | 22 | 5 |
| Energy-recover efficiency[d] ($\Delta H_s/\Delta H_{fus}$) / % | 98 | >99 | 92 |

[a] Eutectic temperature ($T_e$) is $T_m$ of $\mathbf{1}_{0.46}\mathbf{2}_{0.54}$.

[b] $\Delta S_{fus}$ was calculated as $\Delta H_{fus}/T_m$.

[c] Hysteresis is calculated as $T_m - T_c$.

[d] Energy-recover efficiency is calculated as $\Delta H_c/\Delta H_{fus}$.

**Table S5.** Thermal expansion coefficients for $Ag(GN)_2(BF_4)$ (**1**) and $Ag(PN)_2(BF_4)$ (**2**) along their unit cell axis

| No. | Formula | $\alpha_a = \alpha_b$ / ppm °C$^{-1}$ | $\alpha_c$ / ppm °C$^{-1}$ |
|---|---|---|---|
| **1** | $Ag(GN)_2(BF_4)$ | 50 | 61 |
| **2** | $Ag(PN)_2(BF_4)$ | 77 | 90 |

**Table S6.** Eutectic and equilibrium $T_m$ of $\mathbf{3}_x\mathbf{4}_{1-x}$.

| Sample | Eutectic $T_m$ | Equilibrium $T_m$ |
|---|---|---|
| **3** | – | 132 |
| $\mathbf{3}_{0.90}\mathbf{4}_{0.10}$ | 109 | 122 |
| $\mathbf{3}_{0.80}\mathbf{4}_{0.20}$ | 108 | 120 |
| $\mathbf{3}_{0.70}\mathbf{4}_{0.30}$ | 110 | 116 |
| $\mathbf{3}_{0.60}\mathbf{4}_{0.40}$ | 110 | 110 |
| $\mathbf{3}_{0.50}\mathbf{4}_{0.50}$ | 110 | 119 |
| $\mathbf{3}_{0.40}\mathbf{4}_{0.60}$ | 114 | 121 |
| $\mathbf{3}_{0.30}\mathbf{4}_{0.70}$ | 110 | 122 |
| $\mathbf{3}_{0.20}\mathbf{4}_{0.80}$ | 108 | 131 |
| $\mathbf{3}_{0.10}\mathbf{4}_{0.90}$ | 112 | 139 |
| **4** | – | 148 |

**Table S7.** Solvus temperature, eutectic and equilibrium $T_m$ of $\mathbf{3}_x\mathbf{5}_{1-x}$.

| Sample | Solvus temperature | Eutectic $T_m$ | Equilibrium $T_m$ |
|---|---|---|---|
| **3** | – | – | 132 |
| $\mathbf{3}_{0.90}\mathbf{5}_{0.10}$ | – | 127 | 127 |
| $\mathbf{3}_{0.80}\mathbf{5}_{0.20}$ | – | 125 | 132 |
| $\mathbf{3}_{0.70}\mathbf{5}_{0.30}$ | – | 128 | 139 |
| $\mathbf{3}_{0.60}\mathbf{5}_{0.40}$ | – | 128 | 144 |
| $\mathbf{3}_{0.50}\mathbf{5}_{0.50}$ | – | 128 | 153 |
| $\mathbf{3}_{0.40}\mathbf{5}_{0.60}$ | – | 125 | 158 |
| $\mathbf{3}_{0.35}\mathbf{5}_{0.65}$ | 116 | 131 | 160 |
| $\mathbf{3}_{0.30}\mathbf{5}_{0.70}$ | 111 | 137 | 166 |
| $\mathbf{3}_{0.20}\mathbf{5}_{0.80}$ | – | 145 | 173 |
| $\mathbf{3}_{0.10}\mathbf{5}_{0.90}$ | – | 155 | 181 |
| **5** | – | – | 189 |

**Table S8.** Thermochemical parameters of **1**, $\mathbf{1}_{0.75}\mathbf{2}_{0.25}$, $\mathbf{1}_{0.67}\mathbf{2}_{0.33}$, $\mathbf{1}_{0.46}\mathbf{2}_{0.54}$, $\mathbf{1}_{0.20}\mathbf{2}_{0.80}$, and **2**.

| | | $\mathbf{1}_{0.75}\mathbf{2}_{0.25}$ | $\mathbf{1}_{0.67}\mathbf{2}_{0.33}$ | $\mathbf{1}_{0.46}\mathbf{2}_{0.54}$ | $\mathbf{1}_{0.20}\mathbf{2}_{0.80}$ |
|---|---|---|---|---|---|
| Eutectic temperature of melting / ºC | | 100 | 101 | 100 | 100 |
| Equilibrium temperature of melting / ºC | | 131 | 122 | 100 | 110 |
| $\Delta H_{fus}$ / kJ mol$^{-1}$ | Eutectic temperature | 16 | 24 | 40 | 14 |
| | Equilibrium temperature | 13 | 5 | – | 14 |
| | Total | 29 | 29 | 40 | 28 |
| $\Delta H_{fus}$ / kJ kg$^{-1}$ | Eutectic temperature | 40 | 60 | 97 | 33 |
| | Equilibrium temperature | 33 | 13 | – | 34 |
| | Total | 73 | 73 | 97 | 67 |

**Reference**

(S1) Prencipe, M.; Mazzeo, P. P.; Bacchi, A., A method to predict binary eutectic mixtures for mechanochemical syntheses and cocrystallizations. *RSC Mechanochem.* **2025**, *2*, 61-71.
